# Preclinical development of a vaccine against oligomeric alpha-synuclein based on virus-like particles

**DOI:** 10.1371/journal.pone.0181844

**Published:** 2017-08-10

**Authors:** Marika Doucet, Aadil El-Turabi, Franziska Zabel, Benjamin H.M. Hunn, Nora Bengoa-Vergniory, Milena Cioroch, Mauricio Ramm, Amy M. Smith, Ariane Cruz Gomes, Gustavo Cabral de Miranda, Richard Wade-Martins, Martin F. Bachmann

**Affiliations:** 1 Department of Physiology, Anatomy and Genetics, University of Oxford, South Parks Road, Oxford, United Kingdom; 2 The Jenner Institute, Nuffield Department of Medicine, The Henry Wellcome Building for Molecular Physiology, University of Oxford, Roosevelt Drive, Oxford, United Kingdom; 3 Immunology, University Hospital Zürich, Zürich, Switzerland; 4 Immunology, Rheumatology, Immunology and Allergy (RIA), Inselspital, University of Bern, Switzerland; Hertie Institute for Clinical Brain Research and German Center for Neurodegenerative Diseases, GERMANY

## Abstract

Parkinson's disease (PD) is a progressive and currently incurable neurological disorder characterised by the loss of midbrain dopaminergic neurons and the accumulation of aggregated alpha-synuclein (a-syn). Oligomeric a-syn is proposed to play a central role in spreading protein aggregation in the brain with associated cellular toxicity contributing to a progressive neurological decline. For this reason, a-syn oligomers have attracted interest as therapeutic targets for neurodegenerative conditions such as PD and other alpha-synucleinopathies. In addition to strategies using small molecules, neutralisation of the toxic oligomers by antibodies represents an attractive and highly specific strategy for reducing disease progression. Emerging active immunisation approaches using vaccines are already being trialled to induce such antibodies. Here we propose a novel vaccine based on the RNA bacteriophage (Qbeta) virus-like particle conjugated with short peptides of human a-syn. High titres of antibodies were successfully and safely generated in wild-type and human a-syn over-expressing (*SNCA*-OVX) transgenic mice following vaccination. Antibodies from vaccine candidates targeting the C-terminal regions of a-syn were able to recognise Lewy bodies, the hallmark aggregates in human PD brains. Furthermore, antibodies specifically targeted oligomeric and aggregated a-syn as they exhibited 100 times greater affinity for oligomeric species over monomer a-syn proteins in solution. In the SNCA-OVX transgenic mice used, vaccination was, however, unable to confer significant changes to oligomeric a-syn bioburden. Similarly, there was no discernible effect of vaccine treatment on behavioural phenotype as compared to control groups. Thus, antibodies specific for oligomeric a-syn induced by vaccination were unable to treat symptoms of PD in this particular mouse model.

## Introduction

Parkinson’s disease (PD) is the second most common neurodegenerative disorder after Alzheimer’s disease (AD), with current treatments, including pharmacological agents and deep brain stimulation, only offering symptomatic relief [1, 2]. PD is characterised by a loss of neurons in the substantia nigra pars compacta region of the midbrain, leading to a reduction in levels of the neurotransmitter dopamine in the striatum. These events result in the typical motor symptoms observed in PD patients (tremor at rest, rigidity, slowness of movement), while cognitive impairment is usually seen when other brain regions, notably cortical regions, start to be affected [2–5].

Protein inclusions in the brain termed Lewy bodies are the pathological hallmark of PD and other related disorders, such as dementia with Lewy bodies (DLB), and mainly contain aggregated alpha-synuclein [6–9]. Alpha-synuclein (a-syn) is an abundant protein in the brain (representing ~0.5 to 1% cytosolic proteins), is primarily found in the presynaptic terminal of neurons [10, 11] and as little as a 1.5 or 2-fold up-regulation of a-syn expression caused by gene multiplication can cause familial PD [12, 13].

The mechanisms underlying accumulation of a-syn in Lewy bodies are thought to be based on aggregation of a-syn and/or failure to clear a-syn by proteolytic and autophagy pathways [14, 15]. In recent years, studies have also increasingly suggested that aberrant forms of a-syn, including oligomers and fibrils, may interfere with normal cellular processes, promoting further aggregation of protein, leading to spreading of these toxic forms of a-syn from neuron to neuron, and ultimately to neuronal death [16–18]. Notably, one of these reports established a mechanistic link between transmission of one form of pathologic a-syn and the cardinal features of PD [17]. In this study, the authors demonstrated that a single intra-striatal administration of a-syn fibrils led to the cell-to-cell transmission of pathologic a-syn and PD-like pathology in anatomically interconnected brain regions of wild-type mice. In addition, Lewy pathology accumulation in this model resulted in a progressive loss of dopamine neurons in the substantia nigra pars compacta, and was accompanied by reduced dopamine levels culminating in motor deficits [17].

Other forms of a-syn, such as oligomers, may be critical to the pathogenesis of synucleinopathies. Certainly, a-syn oligomers have been detected in the brain of patients affected by synucleinopathies [19–21] and a study demonstrated that the cerebrospinal fluid (CSF) of patients with PD contained increased levels of a-syn oligomers when compared to controls [22]. The presence of extracellular a-syn in oligomeric form raises the possibility of neutralising the oligomers using antibodies as achieved by classical vaccination against infectious diseases. Indeed, monoclonal antibodies (mAbs) targeting a-syn have been shown to reduce spread and templated aggregation of endogenous a-syn by preformed fibrils (PFFs) [23] and related neurotoxic effect in vitro and in vivo [24,25]. A further attractive avenue is active immunisation that utilises a vaccine to induce therapeutic antibody response. Such an approach is in development (by Affiris AG) for AD, PD and multiple system atrophy (MSA) [26–28] and is being trialled in humans. Clinical trials using vaccines targeting a-syn are currently in Phase 1 and focus on safety and tolerability. Reports for PD and MSA thus far indicate that the candidate vaccines targeting a-syn are able to safely induce some levels of antibodies with acceptable tolerability [29,30]. Since the studies are ongoing, no information on secondary outcomes that may relate to efficacy (such as beneficial effect on cognitive and motor symptoms) are available at this time.

The use of virus-like particles (VLPs) as B-cell vaccines to generate high titre antibody responses against numerous biomolecules is increasingly well described [31]. Such vaccines are able to raise antibody responses against self-antigens safely and with excellent tolerability, which make them an ideal tool for targeting aberrant molecules, such as a-syn. This approach has already been successfully applied preclinically in the case of Alzheimer’s disease [32] and Novartis is currently testing this candidate VLP vaccine targeting amyloid beta (CAD106) in a Phase 3 clinical trial (NCT02565511).

Here we describe the preclinical development of a VLP vaccine targeting a-syn. Using a-syn-derived peptides displayed on VLPs, we were able to induce antibodies in mice that recognised Lewy bodies and toxic oligomeric a-syn species with high specificity, while recognition of monomeric a-syn was essentially absent.

## Materials and methods

### Generation of Qb-PD vaccines

Qbeta VLPs were prepared following established methods [33] with minor modifications. In brief, prokaryotic expression vector based on pGEM encoding the Qbeta coat protein (pQb10), was transformed in to *E*. *coli* JM109 cells (Sigma-Aldrich). A starter inoculum in LB-carbenicillin (50 μg/mL) was diluted 10-fold with M9 minimal media with carbenicillin (50 μg/mL), and incubated overnight at 37°C whilst shaking at 220 rpm to induce protein expression. Harvested cells were lysed in 20 mM Tris pH 8.0, 0.1% (v/v) Triton X-100 buffer with Lysonase™ (Merck) at 20 μL/g wet cell pellet weight, and incubated for 1h at room temperature, followed by sonication on ice with 3 cycles (30 s on, 30 s off), at 40% power amplitude. Cell lysates were clarified by centrifugation at 15,000 x g for 30 min at 4°C, and the soluble fraction diluted and subjected to concentration by tangential flow filtration (TFF) step using a 750 kDa molecular weight cut-off (MWCO) hollow-fibre ultrafiltration unit (GE Healthcare). Pump feed was adjusted to maintain the transmembrane pressure (TMP) at ~1 bar during concentration and diafiltration (into column buffer for subsequent purification step). Selective purification by ion exchange chromatography (IEX) using a strong quaternary anion exchanger (TMAE, Merck) separated charged VLPs, from majority of host cell proteins (~90% purity) followed by final size exclusion chromatography (SEC) using Sephacryl S500HR (GE Healthcare), yielding purified homogenous particles.

The short synthetic peptides CGGKNEEGAPQ (PD1), MDVFMKGLGGC (PD2) and CGGEGYQDYEPEA (PD3), respectively representing the middle region, N-terminal and C-terminal sequences of human a-syn (in bold) were selected for vaccine design, the addition of linker residues at the amino or carboxy terminus providing a terminal cysteine residue for efficient conjugation. Of these, the PD1 peptide differs only slightly (by 2 aa) from mouse a-syn, whereas PD2 and PD3 are identical between human and mouse sequences. Moreover, selection of peptides was based on the rationale that these three regions may be more accessible in oligomeric and aggregate forms of a-syn relevant to PD. In addition, the peptide lengths were limited to avoid the possibility of stimulating antigen-specific cellular immunity that may contribute to an undesirable inflammatory response. PD1, PD2 and PD3 were chemically cross-linked onto Qb-based virus-like particles (VLPs) with succinimidyl-6-[(β-maleimidopropionamido)hexanoate] (SMPH) creating Qb-PD1, Qb-PD2 and Qb-PD3 vaccines. Qb (uncoupled to any peptide) was used as negative control.

### Human brain tissue

Tissue samples from patients with PD and control subjects were supplied by the Parkinson’s UK Tissue Bank. Sections of the substantia nigra from one PD patient and one control subject were paraffin-embedded and supplied at 5 μm thick. Sections were prepared as previously described [21]. Paraffin-embedded tissue was dewaxed in xylene and Histo-Clear (National Diagnostics), and rehydrated in graded alcohols. Sections were then blocked in 10% H_2_O_2_ (in PBS) for 1 h at room temperature in the dark to quench endogenous peroxidases. Antigen retrieval was performed in citrate buffer (pH 6.0) by microwave heating for a total duration of 10 min. Tissue was then blocked in 10% normal goat serum in Tris-buffered saline containing 0.1% Triton X-100 (TBS-T) for 1 h at room temperature and incubated in primary antibody diluted in blocking solution overnight at 4°C. The primary antibodies used were: mouse anti-alpha synuclein (SYN211, Abcam) at 1:2000 and purified IgGs from vaccinated mice at 1:1000. Following this, tissue sections were washed and incubated in biotinylated goat anti-mouse antibody (1:200 in blocking solution) (Jackson ImmunoResearch) for 1 h at room temperature, washed again, and incubated in an avidin-biotinylated peroxidase complex formulated in TBS-T (Vectastain ABC Elite, Vector laboratories) for 1h at room temperature. Following a washing step, sections were incubated in a 3,3’-diaminobenzidine (DAB) solution (Sigma) for 3.5 min at room temperature. Finally, the tissue sections were counter-stained with haematoxylin (Vector laboratories) for 5 min at room temperature, dehydrated in an increasing gradient of alcohols and Histo-Clear, before mounting with DPX mounting reagent.

### Mice and experimental design

Wild type mice (C57BL/6J) were purchased from Harlan (now Envigo) or Charles River Laboratories. *SNCA*-OVX mice generated in our laboratory were used in this research project, as previously described [34]. These transgenic mice express wild-type a-syn from the complete human *SNCA* locus at disease-relevant levels on a *Snca*-/- mouse background. Male and female mice aged 2–2.5 months (studies 1, 2 and 3) and 5–5.5 months (study 4) were vaccinated subcutaneously with 20 μg of vaccine (100 μL) every two weeks for a month, followed by monthly injections until the end of the study (**Table 1**). Mice were monitored daily, as well as prior to and following each immunisation and behavioural test. At the end of studies 1 to 4, mice were euthanised using pentobarbitone 20% (w/v) (100 μL or 20 mg per mouse, i.p.) and transcardially perfused with PBS (pH 7.4). All procedures were conducted in accordance with the UK Animals (Scientific Procedures) Act of 1986 and approved by the Animal Welfare and Ethical Review Bodies at the Department of Physiology, Anatomy and Genetics and the Nuffield Department of Clinical Medicine, University of Oxford **(S1 Checklist)**.

**Table 1:** Age of mice and duration of immunisations for each experimental study group.

Name of study	Age at start of study	Duration of immunisation protocol	Groups
Study 1	2–2.5 months	2 months	PBS (n = 5), Qb (n = 6), Qb-PD1 (n = 6) and Qb-PD3 (n = 6)
Study 2	2–2.5 months	3 months	PBS (n = 4), Qb (n = 4), Qb-PD1 (n = 4) and Qb-PD3 (n = 4)
Study 3	2–2.5 months	4 months	PBS (n = 4), Qb (n = 4), Qb-PD1 (n = 4) and Qb-PD3 (n = 4)
Study 4	5–5.5 months	13 months	PBS (n = 14), Qb (n = 14), Qb-PD1 (n = 13) and Qb-PD3 (n = 16)

### Antibody titres

Antibody titres were monitored by ELISA analysis. Mice were bled at d0, d14, d28 and monthly thereafter (until the end of the study). Blood was collected in Microvette CB300 tubes containing a clotting activator (Sarstedt) and centrifuged at 10,000 x g for 5 min at room temperature. The serum fraction was isolated and frozen at -20°C until further use. For antibody titre determination, microplates were coated with either the RNase-conjugated with peptide (respectively used for immunisation) at 7 μg/mL, recombinant full-length human alpha-synuclein commercially sourced (rPeptide) or prepared as described below, or recombinant full-length beta-synuclein (b-syn) (rPeptide) at 1 μg/mL. Samples were blocked for 2 h at room temperature in 2% BSA/PBS-T, followed by incubation in serum (serial dilutions in 2% BSA/PBS-T) for 2 h at room temperature. Following washing, samples were incubated in goat anti-mouse HRP antibody (1:2000, Sigma) diluted in PBS-T for 1 h at room temperature. Samples were then washed again, before being incubated in TMB substrate (Sigma) for 10 min in the dark. The reaction was stopped with 0.15–0.5 M H_2_S0_4_ and plates were read at 450 nm. Antibody titres were determined at half maximal signal (OD50).

### Antibody affinity estimation

Vaccine-induced antibody affinities for respective peptides were estimated by competition ELISA analysis of collected sera. Microplates were coated with RNase-coupled peptides reflecting those used for immunisation at 7 μg/mL, or recombinant full-length human a-syn (prepared as described below) at 1 μg/mL. Microplates were blocked for 2 h at room temperature in 2% BSA/PBS-T, followed by incubation with serum IgG that had been pre-incubated in solution for 1 h at room temperature with increasing concentrations of free peptide or free recombinant protein (in 2% BSA/PBS-T). This mixture was applied to plates for 2 h at room temperature. Samples were processed as described above for ELISA to develop signal. The dissociation constant Kd was estimated as the concentration of competing peptide or protein that lead to 50% decrease in absorbance at 450 nm.

### Immunoblot analysis

Hemibrains were homogenised in RIPA buffer (SDS-PAGE) or non-denaturing lysis buffer (native PAGE), as previously described [34]. For immunoblot analysis, 10 μg of total protein per lane was loaded on 4–15% gels (Bio-Rad) and blotted onto polyvinylidene difluoride membranes (Bio-Rad). To determine the effects of vaccination on the levels of alpha-synuclein, MHCII and Fc-gamma receptor, blotted samples from immunised *SNCA*-OVX mice were probed with the rabbit MJFR1 antibody against human a-syn (Abcam, 1:1000), MHCII (eBioscience, 1:500) and Fc-gamma receptor CD16/32 (Abcam, 1:500). Overnight incubation at 4°C was followed by incubation in goat anti-rabbit secondary HRP antibody (Bio-Rad, 1:5000) for 1–2 h at room temperature, and visualisation with enhanced chemiluminescence (Millipore). Beta-actin was used as a loading control. To examine which species of a-syn are recognised by antibodies produced after immunisation, 0.1 μg of recombinant a-syn and a-syn oligomers were loaded on 4–15% SDS-PAGE gels and analysed by immunoblot using vaccination-elicited antibodies as primary antibody (1:500) and goat anti-mouse HRP antibody (Bio-Rad, 1:5000) as secondary antibody. The monoclonal antibody SYN211 (Abcam, 1:5000) served as positive control.

### Preparation of alpha-synuclein protein and oligomers

Human a-syn coding sequence (1-140aa from UniProtKB number: P37840-1) preceded by a hexahistidine tag with a tobacco etch virus (TEV) protease cleavage site, was codon optimised for *E*. *coli* and synthesized with flanking restriction sites (GeneArt, ThermoFisher). This coding region was inserted by restriction enzyme double digest into a prokaryotic expression vector (pET28b, Novagen). The resultant plasmid was transformed in to an *E*. *coli* expression host (BL21 Star (DE3), ThermoFisher) and protein induced with 1 mM IPTG (Sigma) at 37°C, for 4 h. Clarified soluble cell lysate was subjected to Nickel-ion affinity purification via His-tag (HisTrap™ Excel, GE Healthcare) following manufacturer’s instructions. This was followed by overnight TEV cleavage at 4°C for removal of the His-tag and further affinity chromatography, yielding approximately 90% pure a-syn. Alpha-synuclein oligomers were prepared from purified a-syn as previously described [21, 35]. Briefly, oligomers were produced by incubating purified recombinant human a-syn (or commercially sourced from rPeptide) at 1 mg/mL (70 μM) with a 30:1 molar ratio excess of 4-hydroxy-2-nonenal (HNE) (Cayman Chemicals) for 18 h at 37°C. Following incubation, unbound aldehyde was removed using an Amicon Ultra 3 kDa cut-off centrifugal filter unit (Millipore).

### Negative-stain electron microscopy

Aliquots of purified Qb VLP or a-syn samples were diluted to 0.2 mg/mL and 0.5 mg/mL, respectively, then deposited onto glow-discharged, carbon-coated Formvar copper grids (Electron Microscopy Sciences). After a 30 s incubation, the excess sample was blotted away, and the grids were washed twice with deionized water. Samples were stained with 2% (w/v) uranyl acetate for 45 s, and excess stain was removed by blotting. Dried grids were examined on a Tecnai T12 transmission electron microscope operated at 80 kV. Images were acquired on a 4,000 × 4,000 high-sensitivity FEI Eagle camera typically at 52,000x – 67,000× magnification, the latter of which corresponded to 1.68 Å/pixel sampling of the specimen.

### ELISA analysis of a-syn protein levels

Human a-syn protein levels from *SNCA*-OVX brain samples were measured by ELISA. Hemibrains were homogenised in 5 M guanidine HCl/50 mM Tris HCl solution (pH 8.0) for 4 h at room temperature. Samples were then diluted (1:50 to 1:600) in cold reaction buffer (5% BSA and 0.03% Tween-20 in DPBS) and centrifuged at 16,000 x g for 20 min at 4°C. Supernatants were carefully decanted and stored on ice until used with the a-syn ELISA kit (Invitrogen) following the guidelines provided by the manufacturer.

### Preparation of mouse spleen tissue

Mice were anaesthetised with pentobarbitone 20% (w/v) (100 μL or 20 mg per mouse, i.p.) and transcardially perfused with PBS (pH 7.4), followed by 4% paraformaldehyde (PFA) (v/v). Spleens were post-fixed in 4% PFA for at least 24h at 4°C, paraffin-embedded and 5 μm sections were cut for haematoxylin/eosin (H&E) staining.

### Fluorescence immunohistochemistry for paraffin-embedded mouse tissue

Mice were anaesthetised with pentobarbitone 20% (w/v) (100 μL or 20 mg per mouse, i.p.) and transcardially perfused with PBS (pH 7.4), followed by 4% paraformaldehyde (PFA) (vol/vol). Brains were post-fixed in 4% PFA for one week at 4°C, stored in 70% ethanol at 4°C until samples were paraffin-embedded and 5 μm sections were cut. Paraffin-embedded brain tissue was prepared for immunohistochemistry by dewaxing in xylene and Histo-Clear, rehydrating in graded alcohols, and retrieval of antigens in citrate buffer (pH 6.0) and microwave heating for 10 min with 5 min breaks. Tissue sections were washed in 0.01 M PBS and blocked in normal goat serum for 2 h at room temperature. For microglia staining, sections were incubated in rabbit anti Iba-1 antibody (1:750, Wako) overnight at 4°C, incubated in biotinylated anti-rabbit IgGs (1:200, Vector laboratories) for 1 h at room temperature, washed in TBS-T (0.05% Tween) and blocked in 10% normal goat serum (containing 1 M glycine TBS and 0.1% Triton X-100). The primary antibodies used were mouse anti sheep anti a-syn (1:500, Abcam), and rabbit anti Iba-1 (1:500, Wako). Goat anti-mouse 680nm IgG (H+L) (1:500 Life Technologies) was used to detect mouse IgG (H+L). The next day, sections were washed in TBS-T and incubated with appropriate Alexa fluor goat anti-sheep 488 nm and goat anti-rabbit 594 nm secondary antibodies diluted in TBS-T (1:200, Life Technologies) for 1h at room temperature. For nuclear staining, sections were incubated in 4’,6-diamidino-2-phenylindole (DAPI) (1:2000, Life Technologies) for 10 min at room temperature. Sections were mounted with FluorSave mounting medium (Calbiochem) before being visualised with an Evos FL auto imaging system (Life Technologies).

### Alpha-synuclein proximity ligation assay (AS-PLA)

Mice were anaesthetised with pentobarbitone 20% (w/v) (100 μL or 20 mg per mouse, i.p.) and transcardially perfused with PBS (pH 7.4), followed by 4% paraformaldehyde (PFA) (v/v). Brains were post-fixed in 4% PFA for one week at 4°C, stored in 70% ethanol at 4°C until samples were paraffin-embedded and 5 μm sections were cut. Alpha-synuclein proximity ligation assay (AS-PLA) experiments were carried out on brain sections, using Duolink® kits (Olink Bioscience) for brightfield or fluorescent dyes. The a-syn antibody chosen for the AS-PLA probes was SYN211 (Abcam). Brightfield AS-PLA was carried out as previously described [21]. For PLA co-immunofluorescence, sections were de-waxed in xylene and Histo-Clear, peroxidase reaction blocked in 0.3% H_2_O_2_ for 30 min at room temperature, and antigen retrieved in citrate buffer pH 6 (Abcam 93678), after which brain sections were blocked in 10% normal goat serum (containing 1 M glycine TBS and 0.1% Triton-X100) and incubated for 1h in primary antibody (anti-TH at 1:500) (ab152, Millipore). Sections were then washed with TBS containing 0.1% Triton-X100 and incubated for 1h in the dark with Alexa Fluor 488 secondary antibody (Life Technologies). All samples were washed in TBS + 0.05% Tween 20 (TBS-T) and incubated in Duolink® block solution for 1h at 37°C, followed by overnight incubation with the conjugates diluted in Duolink® PLA diluent (1:100) at 4°C and subsequent PLA steps as described previously [21].

All fluorescent images were acquired with an EVOS FL auto imaging system (Life Technologies) at 20 x magnification and were automatically analysed with ImageJ for counting total PLA puncta. Intracellular PLA puncta were quantified manually after masking for TH positive cells. All image acquisition and counting were done blind. For each area, 4 random images were taken and analysed in order to provide a representative sampling of the tissue. Counts are expressed as average PLA puncta per imaging field or positive cell.

### Free-floating immunofluorescence staining of mouse tissue

Mice were anaesthetised with pentobarbitone 20% (w/v) (100 μL or 20 mg per mouse, i.p.) and transcardially perfused with PBS (pH 7.4), followed by 4% paraformaldehyde (PFA) (v/v). Brains were post-fixed in 4% PFA for 24 h, cryoprotected in 30% sucrose for 72 h before 35 μm sections were cut. Free-floating sections were stored at -20°C in anti-freeze solution (50% PBS, 25% ethylene glycol, 25% glycerol) until staining was performed. Sections were washed in PBS, blocked in 10% normal goat serum for 1 h at room temperature and incubated in primary antibodies diluted in blocking solution overnight at 4°C. The primary antibodies used were mouse anti a-syn (1:500, BD Biosciences), and rabbit anti-tyrosine hydroxylase (TH) (1:500, Millipore). The next day, sections were washed in PBS containing 0.1% Triton-X100 (PBS-TX) and incubated with appropriate Alexa fluor goat anti-mouse 488 nm and/ or goat anti-rabbit 594 nm secondary antibodies diluted in PBS-T (1:200, Life Technologies) for 1h at room temperature. For nuclear staining, sections were incubated in 4’,6-diamidino-2-phenylindole (DAPI) (1:2000, Life Technologies) for 10 min at room temperature. Sections were mounted with FluorSave mounting medium (Calbiochem) before being visualised with an Evos FL auto imaging system (Life Technologies).

### Behavioural testing of mice

#### Animal housing

Mice were group-housed in a 12 h-12 h light-dark cycle with the lights on at 07.00. All experiments were conducted during the light cycle. Food and water were available ad libitum. Body weight was assessed monthly as a gross measure of health following administration of Qb-PD vaccines.

#### Rotarod

Mice were placed on a rod that accelerated from 4 to 40 rpm over a 5-min period. Mice were trained in sessions consisting of three trials of 5 min for three consecutive days, followed by testing on the fourth day (three trials of 5 min). The latency to fall was recorded and performance was averaged for each day [34, 36].

#### Locomotor activity

Mice were placed into activity monitor cages and locomotor activity was recorded for 4 h using the photobeam activity system-home cage (PAS-HC, San Diego Instruments). Each activity monitor was equipped with a set of horizontal infrared beams, positioned above the base of the cage. Activity was measured as the number of times a beam changed from unbroken to broken.

#### Digitised gait assessment

Mice were subjected to gait assessment using the CatWalk automated gait analysis system (Noldus Information Technology). Briefly, mice were placed on a transparent glass platform cross-illuminated by a green light emitting diode. Video recordings of mice ambulating across the platform were made, and were accepted for analysis if they were between 0.5 and 5 seconds in duration and there was less than 35% variation in speed. The first 5 compliant runs were analysed and averaged, or in the event of the mouse making 100 runs without reaching this target, all of the compliant runs to this point were analysed. A pre-determined set of gait parameters relating to parkinsonism (forelimb and hindlimb stride length and swing speed, and gait velocity and cadence) were analysed.

#### Gastro-intestinal function (one-hour stool collection)

Mice were placed into separate clean cages and faecal pellets were collected over a 1 h period (16.00–17.00), as described previously [34]. Pellets were weighed to obtain wet stool weight, dried overnight at 65°C, and reweighed to obtain dry stool weight and calculate stool water content (water content = wet stool weight—dry stool weight).

#### Inverted screen test

This test measures muscle strength and is used to screen for strength deficits that may confound tests of motor function. Mice were placed in the centre of a 50 cm x 50 cm wire grid framed by a 4 cm wooden frame, with a mesh of 12 mm squares of 1 mm in diameter. The grid was then immediately inverted with the head of the mouse declining first and held 50 cm above a cushioned surface. The latency to fall was recorded with a maximal trial time of 60 s [34, 37].

#### Statistical analysis

Data are expressed as mean ± standard error of the mean (SEM) and were analysed using analyses of variance (assuming normal distribution of data). If any statistically significant change was found following one- or two- factor analysis of variance, post hoc comparisons were performed using Dunnett’s, Dunn’s or Tukey’s tests, where appropriate. Data were deemed significant when P<0.05.

## Results

### Generation of vaccines

**Fig 1:**
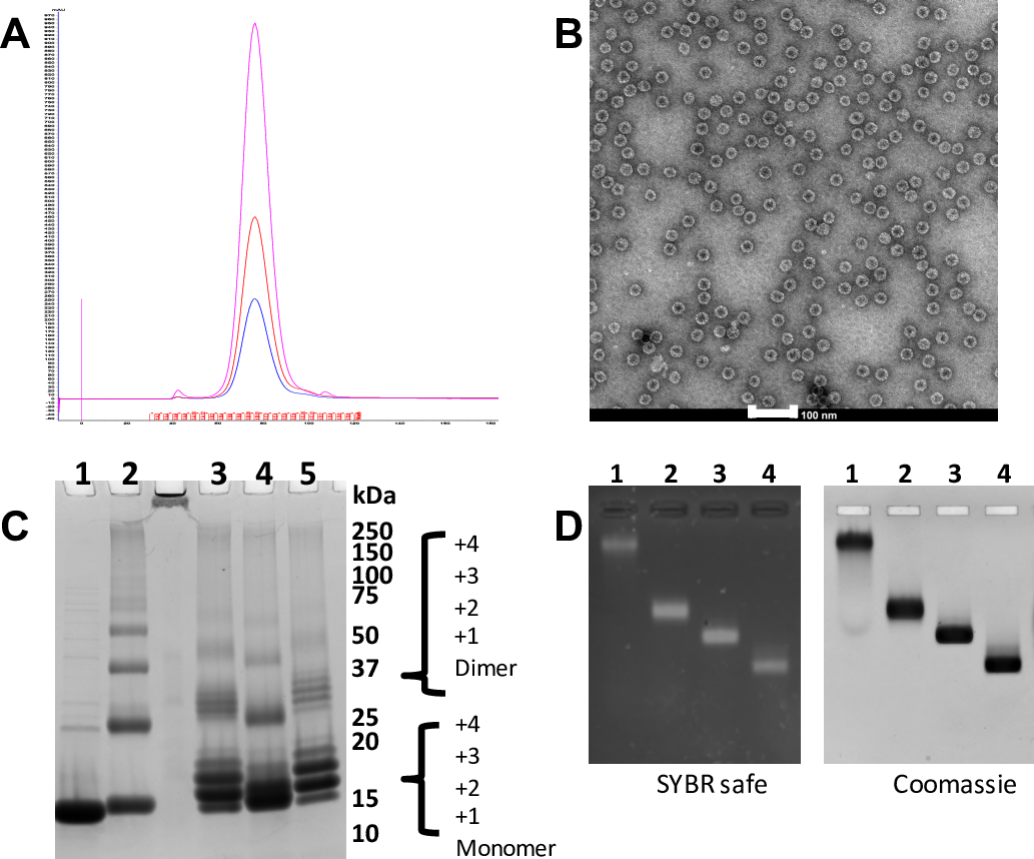
Qb VLP and PD vaccine preparation. (A) Qb positive fractions from anion exchange were pooled and concentrated prior to size exclusion chromatography (SEC) on 16/600 Sephacryl S500-HR column, elution monitored by UV at 280 nm (blue), 254 nm (red) and 220 nm (pink). (B) A sample from the major SEC peak (adjusted to 0.1 mg/mL) was negatively stained and viewed by TEM (scale bar, 100nm). (C) Coomassie stained SDS-PAGE of peptide conjugated VLP preparation of PD vaccines. Purified VLPs (lane 1), derivatised with SMPH (lane 2) and subsequent conjugation with peptides PD1 (lane 3), PD2 (lane 4) and PD3 (lane 5) (Precision Plus Protein Standards, Bio-Rad. Sizes in kDa as indicated). (D) Vaccine preparations loaded on native agarose gel were stained for nucleic acid with SYBR safe (left) and for protein with Coomassie (right). Purified VLPs (lane 1), derivatised with SMPH (lane 2), conjugated with peptides PD1 (lane 3) and PD3 (lane 4).

The Qbeta (Qb) bacteriophage coat protein interacts with RNA and spontaneously forms VLPs when expressed in *E*. *coli*. These can be used to effectively present antigens to immune effector cells and stimulate strong humoral responses. Qb VLPs were expressed in *E*. *coli* and purified to homogeneity by anion exchange and size exclusion chromatography **(Fig 1A),** a single major peak with absorbance at 254 nm greater than at 280 nm is typical for Qb, indicating encapsidated RNA, and purity >90%. Transmission electron microscopy confirmed purification of regular particles with approximately 30 nm diameter **(Fig 1B)**. To allow coupling of a-syn peptides to Qb VLPs, a cysteine (Cys) was added at one of the termini of the peptides. Peptides were chemically coupled to Qb VLPs following established protocols using SMPH, thereby creating Qb-PD1, Qb-PD2 and Qb-PD3 vaccines. Qb (uncoupled to any peptide) was used as negative control. Coupling was confirmed by visualising additional bands on denaturing gels with masses corresponding to Qb monomer (or higher-order intermediates) linked to 1 and up to 4 peptides **(Fig 1C)**. Native gels revealed that peptide-conjugated particles are intact **(Fig 1D)**, as RNA remains encapsidated and their increased electrophoretic mobility is indicative of increased surface charge conferred by successful decoration of cross-linker and subsequently with peptides.

### Immunisation with vaccines safely induce antibodies against a-syn

**Fig 2:**
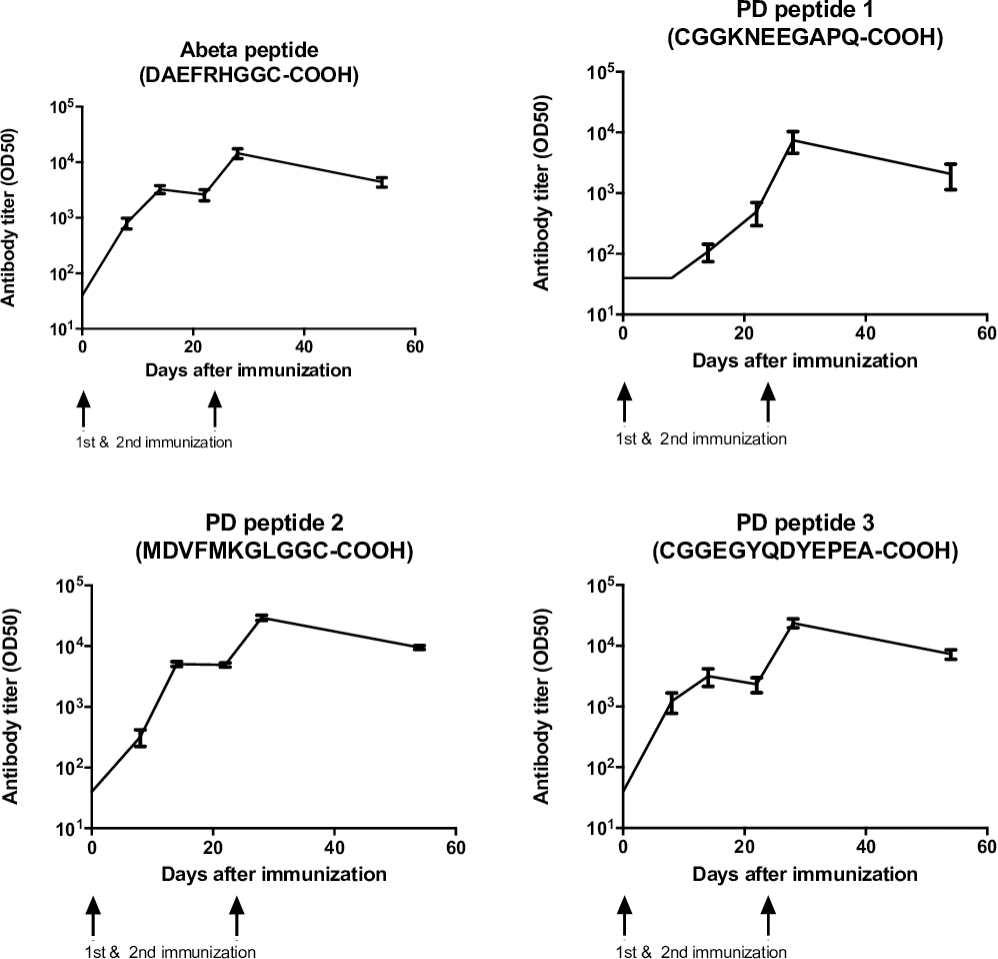
Peptide-specific antibody responses induced by Qb-PD vaccines. Male and female WT C57BL/6 mice received 20 μg of Qb-PD1, Qb-PD2, Qb-PD3 or Aβ_1–6_ (positive control) intravenously at d0 and d21. Antibody titres were determined using ELISA and are expressed as mean ± SEM (n = 4 mice per group).

To examine the immunogenicity of Qb-PD vaccines, male and female WT C57BL/6 mice were immunised with 20 μg of Qb-PD vaccines at day 0 (d0) and d21. Experimental groups were as follows: Qb-PD1 (n = 4), Qb-PD2 (n = 4) and Qb-PD3 (n = 4). Blood was collected at regular intervals until d70 to establish the kinetics of antibody production and to characterise the ability of the produced antibodies to recognise a-syn. A vaccine targeting Aβ_1–6_ (CAD106, a biosimilar of Novartis currently in phase 3 for the treatment of AD) was included for comparison (n = 4). Immunisation of these mice generated excellent titres following prime and booster injections (OD50 10^3^−10^4^), comparable to the AD vaccine candidate based on a similar strategy **(Fig 2)**. Similar results were observed using the intravenous (**Fig 2**) and subcutaneous (s.c.) routes of immunisation **(S1 Fig)**.

### Antibodies produced following immunisation with Qb-PD vaccines recognise monomers, oligomers and aggregates of a-syn

**Fig 3:**
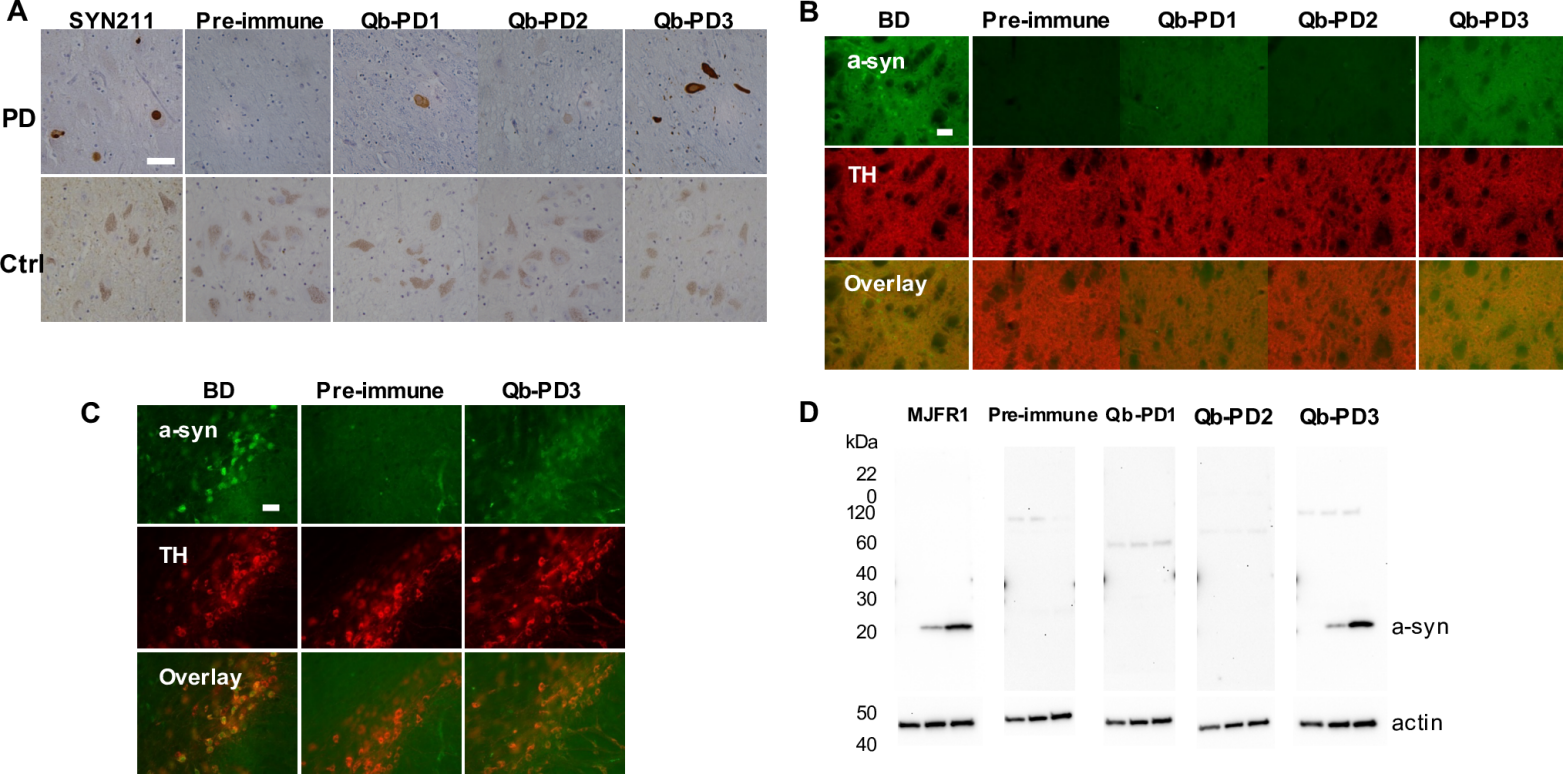
Recognition of a-syn in human and mouse brain tissue. (A) Immunohistochemistry on paraffin-embedded brain tissue from a PD patient and control individual. Purified IgGs (1 mg/mL) used at 1:1000, commercial SYN211 antibody used at 1:2000. Region sampled, substantia nigra. Immunofluorescence on free-floating sections from the (B) striatum and (C) substantia nigra of 3 month old *SNCA*-OVX mice. Purified IgGs (1 mg/mL) used at 1:250, commercial BD a-syn antibody used at 1:500. Scale bars, 50 μm. (D) WB on 20 μg of striatum brain lysate from 3 month old mice. Samples from *Snca-/-* mice, mice over-expressing a-syn at moderate levels (line 21) and mice over-expressing a-syn with a two-fold increase (*SNCA*-OVX) were loaded (left-right). The MJFR1 antibody was used at 1:1000, while IgGs (1 mg/mL) from vaccinated mice were used at 1:500. Actin was used a loading control.

Having determined that mice immunised with Qb-PD vaccines produced antibodies against a-syn peptides, the ability of these antibodies to recognise physiologically relevant a-syn species in human post-mortem tissue from PD patients was assessed. Immunohistochemistry on brain tissue from a PD patient (Braak stage 4) using a commercial anti a-syn antibody showed Lewy bodies and Lewy neurites containing aggregated a-syn in the substantia nigra, as previously described [6]. More interestingly, whole IgG fractions obtained from sera of vaccinated mice also recognised Lewy bodies and Lewy neurites in the substantia nigra of the same patient. Vaccine candidate Qb-PD3 was the most efficient at revealing a-syn aggregates, followed by Qb-PD1. In contrast, Qb-PD2 failed to detect Lewy bodies and Lewy neurites. As expected, IgGs prior to immunisation (from pre-immune or day 0 sera) did not recognise these structures. In paraffin-embedded sections of brain tissue of a control individual without PD, numerous pigmented cells were observed (due to intracellular neuromelanin) but no a-syn was stained **(Fig 3A)**. Taken together, these data suggest that the antibodies produced following immunisation with Qb-PD3 and Qb-PD1 specifically recognise aggregated human a-syn as found in the brain of a PD patient but not non-aggregated a-syn in sections from a control patient.

Next, purified IgGs from vaccinated mice were used for immunofluorescent staining of brain tissue from 3-month old *SNCA*-OVX mice **(Fig 3B)**. Using a commercial antibody, a-syn was found to colocalise with dopaminergic neurons identified by immunostaining for tyrosine hydroxylase (TH), in line with previous work [34]. The detection of a-syn was then assessed using purified IgG from vaccinated mice. Pre-immune IgGs were used as negative control and as expected, pre-immune IgGs did not recognise a-syn. Interestingly, in the striatum, IgGs from Qb-PD3 vaccinated mice were able to detect a-syn, to a similar degree as the commercial anti a-syn antibody. IgGs from Qb-PD1 were also able to recognise a-syn in striatum sections, but to a lesser extent. In contrast, IgGs from Qb-PD2 again did not lead to any detectable signal. When examining the substantia nigra, only IgGs from Qb-PD3 treated mice were able to recognise a-syn **(Fig 3C)**, while IgGs from Qb-PD1 and Qb-PD2 vaccinated mice were unable to detect a-syn (**S2 Fig**).

Consistent with data obtained by immunofluorescence, western blot (WB) experiments showed that pre-immune IgGs did not recognise a-syn in brain homogenates. IgGs from Qb-PD3 vaccinated mice recognised a-syn monomers (1 band at ~20 kDa), while IgGs from Qb-PD1 treated mice did not detect a-syn monomers at 1:500 **(Fig 3D)**. Of interest, WB experiments allow bivalent binding of antibodies, allowing even low affinity antibodies to recognise proteins (see below). Again, IgGs from Qb-PD2 vaccinated mice were not able to recognise a-syn monomers. This is not surprising since the same IgGs were unable to detect a-syn and Lewy bodies in mouse and human brain sections, respectively. In view of these results, the Qb-PD2 vaccine was discontinued, in favour of the leading vaccine candidates Qb-PD1 and Qb-PD3.

**Fig 4:**
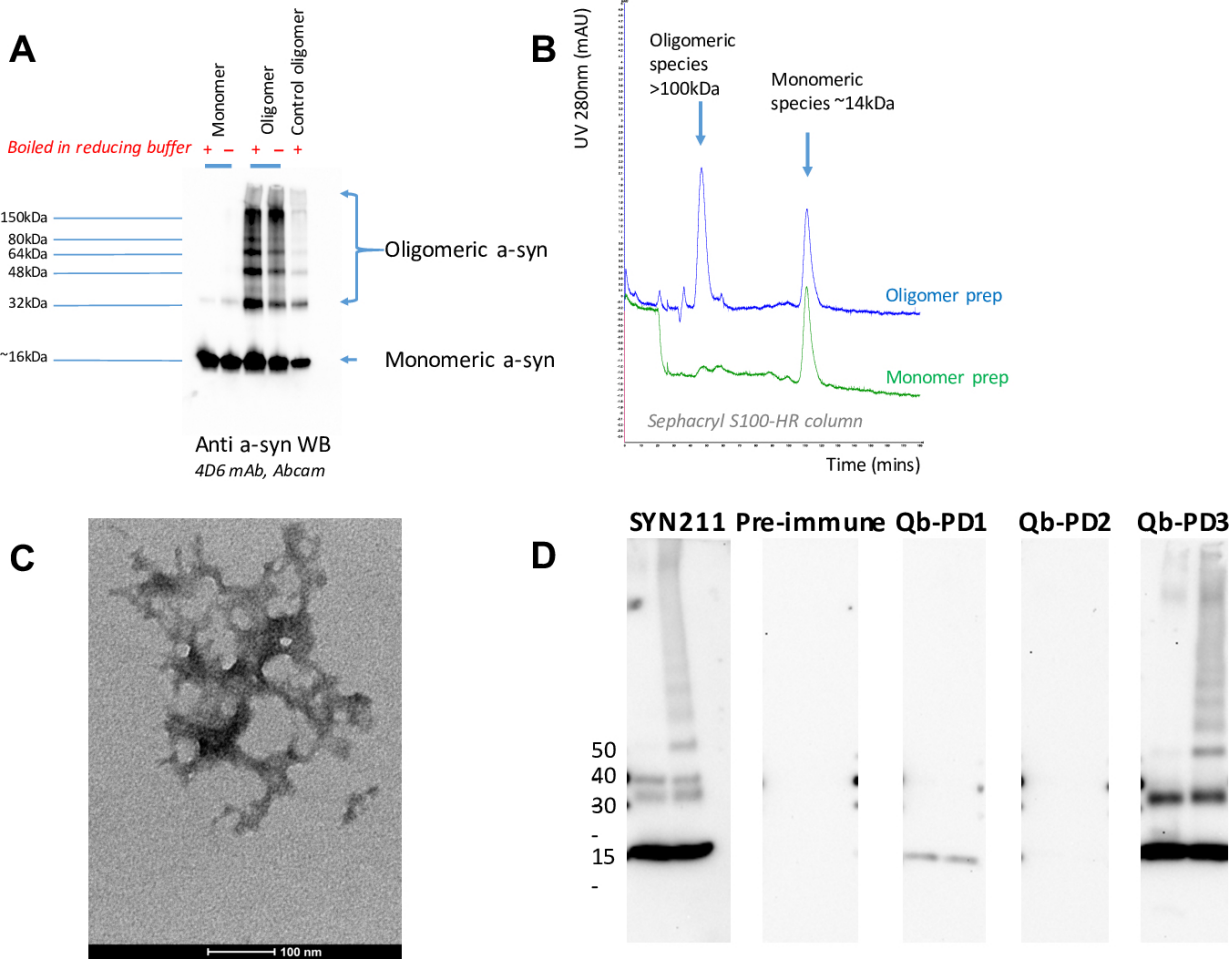
Characterisation of a-syn oligomer preparations and recognition of these oligomers by IgGs from Qb-PD vaccinated mice. (A) Western blot (WB) of monomer and oligomer a-syn preparations reduced with (+) or without heating (-) probed with 4D6 a-syn antibody (1:2000). (B) A 100 μL sample of each preparation was passed through a Sephacryl S100-HR column, displaying over-layered chromatograms to compare UV (280nm) elution profiles. (C) Negative stained TEM of tangled oligomer preparation (scale bar, 100nm). (D) WB on 0.1 μg of full-length recombinant a-syn monomers (left lane) and a-syn oligomers prepared with 4-hydroxy-2-nonenal (HNE) (right lane). WB were probed using SYN211 a-syn antibody (1:5000) and IgGs (1:500) from vaccinated male and female *SNCA*-OVX mice. These vaccinated mice received 20 μg of Qb, Qb-PD1 or Qb-PD3 every two weeks for a month, followed by a monthly injection (total duration of immunisation: 2 months). Pre-immune refers to sera of mice collected prior to first immunisation at d0.

To determine whether purified IgGs from vaccinated mice recognised oligomeric a-syn, oligomers were prepared in vitro using HNE [30] before being loaded in an SDS-PAGE gel. First, characterisation of these oligomer preparations using a commercial antibody for WB revealed apparent regular banding intermediates up to 150 kDa (**Fig 4A**). Size exclusion chromatography under native conditions produced 2 peaks and suggested they comprised mainly of monomers and larger order species (>100 kDa) **(Fig 4B)**, while electron microscopy detected small clumped and tangled aggregates that varied between approximately 100–200 nm in diameter **(Fig 4C)**. This agreed with previous observations [28] and suggested production of small oligomers that were representative of early aggregate intermediates. These oligomers were then loaded in a SDS-PAGE gel. IgGs from Qb-PD1 treated mice only detected a-syn monomers (band at 20 kDa), while IgGs from Qb-PD3 vaccinated mice recognized a-syn monomers as well as a-syn oligomers **(Fig 4D)**.

### Biochemical determination of relative affinities of antibodies

**Fig 5:**
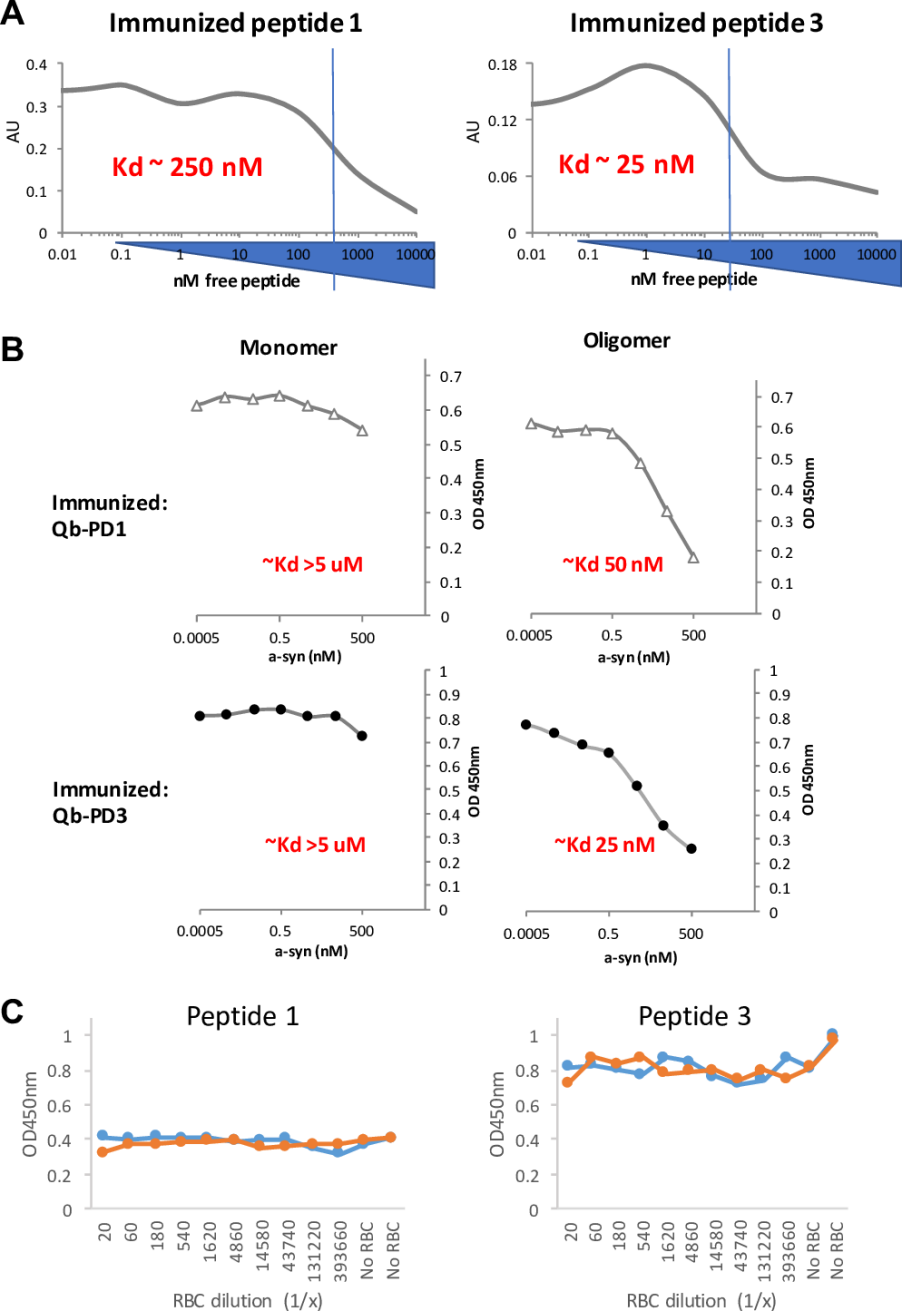
Competition ELISA to estimate relative affinity of vaccine-induced antibodies. Antigens in the form of peptides or recombinant monomer/oligomer protein preparations as described in the methods were coated on to microtitre plates and ELISA reading measured with fixed concentrations of serum IgG antibodies (at OD50 dilution) purified from pooled sera of vaccinated mice (n = 4), preincubated with serial dilutions of (A) free a-syn peptides (PD1, left; PD3, right); (B) free a-syn protein, either monomeric (left panels) or oligomeric (right panels) species, PD1 (top) and PD3 (bottom); and (C) liberated a-syn from haemolysed RBCs (orange line) or PBS negative control (blue line) for PD1 (top) and PD3 (bottom).

In light of the different pattern of recognition of a-syn oligomers by IgGs from Qb-PD1 and Qb-PD3 vaccinated mice described above, further biochemical experiments were carried out to better understand these differences. We next measured relative affinities of the antibodies for their antigen (peptide and protein). To this end, IgGs were competed against increasing concentrations of soluble free peptides in a competition ELISA **(Fig 5A)**. Competition with respective free peptide (same as used in immunisation) led to a reduction in the measured signal and the concentration at which the signal is reduced by half correlates with relative affinity (Kd) of the antibody for its antigen which was in the range of 10^−7^ M. Typically, high affinity (Kd <5 nM) is required for biological activity of antibodies. Therefore, the affinities measured here against soluble peptides are quite modest, suggesting that the antibodies raised are unlikely to be effective in neutralising soluble monomeric forms of a-syn.

Regular indirect ELISA is unable to differentiate between monomer and oligomer species as they essentially resemble similar aggregates when coated directly on to microtitre plates. Therefore, to investigate binding properties of the antibodies against the native monomer versus oligomer species in solution, the use of an in vitro competition ELISA with recombinant oligomeric a-syn was required. For both Qb-PD1 and Qb-PD3 vaccines the monomer proteins displayed very poor competition for binding to the antibodies, with the Kd estimated to be >5 μM. Juxtaposed to this was the result from the oligomeric preparation that appeared to have Kd estimates of 25–50 nM, representing 100 to 200-fold greater affinity of the antibodies for oligomers over the monomers **(Fig 5B)**. If concentrations are corrected for the fact that monomers are the dominant species in the oligomer preparation, apparent affinities/avidities are in the low nanomolar range. This impressive selectivity strongly suggests that these antibodies would fail to bind soluble monomers and exhibit a preference for oligomers in solution.

Given the high amounts of a-syn typically found in red blood cells (RBCs), a further concern was that inadvertent haemolysis during blood sampling could interfere with antibody titre quantification. In an effort to address this question excess a-syn liberated from lysed RBCs were used to spike sera from vaccinated mice to test for interference. The results demonstrated that excess liberated a-syn from *SNCA*-OVX mice failed to interfere with antibodies from either vaccine for recognition of their cognate epitopes on their respective peptides. This was observed equally in both WT C57BL/6 mice and transgenic (*SNCA*-OVX) mice, and similarly in unrelated responses (e.g. antibodies against the VLP carrier) **(Fig 5C)**. These data further support the conclusion that monomeric a-syn is poorly recognised by the induced antibodies.

Taken together, these results indicate that Qb-PD vaccination has the potential to neutralise and/or eliminate putative neurotoxic oligomers and aggregated intermediates, without overtly disrupting the biological function of native monomers. However, it remained to be demonstrated whether active immunisation with Qb-PD vaccines was safe in mice and whether it would bring any physiological benefits in immunised mice over-expressing human a-syn.

The *SNCA*-OVX mouse model expressing high levels of human a-syn on a mouse *Snca-/-* background to avoid the confounding effect of mouse a-syn developed in our laboratory was chosen to assess the safety, tolerability and efficacy of Qb-PD vaccines following short and long term immunisation protocols. *SNCA*-OVX mice exhibit early-onset circuit-specific deficits in dopamine neurotransmission followed subsequently by alterations in neuronal firing properties, a motor phenotype and neuron loss in the absence of overt protein aggregation pathology in the substantia nigra and striatum [34].

In order to obtain an estimation of safety and tolerability of our vaccination approach, the body weight of mice was monitored and physical appearance examined over the time course of all studies (short and long term). All treatment groups demonstrated a physiological increase in weight during the treatment protocol and we observed no evidence of vaccine-mediated adverse effects. A typical example of healthy mice gaining weight is given for study 1 **(S3A Fig).**

Since a-syn is expressed at high levels in erythrocytes [38, 39], blood and spleen samples from experimental mice were analysed to search for potential adverse effects at the end of study 1. Whole blood from vaccinated mice was analysed to examine the safety of Qb-PD vaccine responses on blood parameters (total blood counts performed by Clinical Pathology Laboratory, MRC Hartwell (Swindon, UK) and Diagnostic Laboratories, The Royal Veterinary College (Hatfield, UK)). No obvious differences were found between the groups (data not shown).

In addition to its immunological function, the spleen filters blood and removes old and damaged erythrocytes [40, 41]. The spleen of vaccinated mice was therefore examined for signs of abnormality at the end of study 1, and histology was carried out to verify that Qb-PD vaccines did not cause unwanted effects on spleen regions rich in red blood cells (red pulp). The spleen of Qb-PD treated mice was normal (elongated, dark-red) and not enlarged at the end of the study **(S3B Fig)**. Histological analysis of the spleen did not reveal any gross abnormality of the red pulp of the spleen, further suggesting that a-syn potentially recognised by the induced antibodies in red blood cells was not causing damage after immunisation with Qb-PD1 or Qb-PD3 **(S3C Fig)**. In summary, immunisation of WT and *SNCA*-OVX mice with Qb-PD vaccines for 2 months was safe and well tolerated, based on blood parameters and spleen analysis, with no noticeable differences between treatment groups.

**Fig 6:**
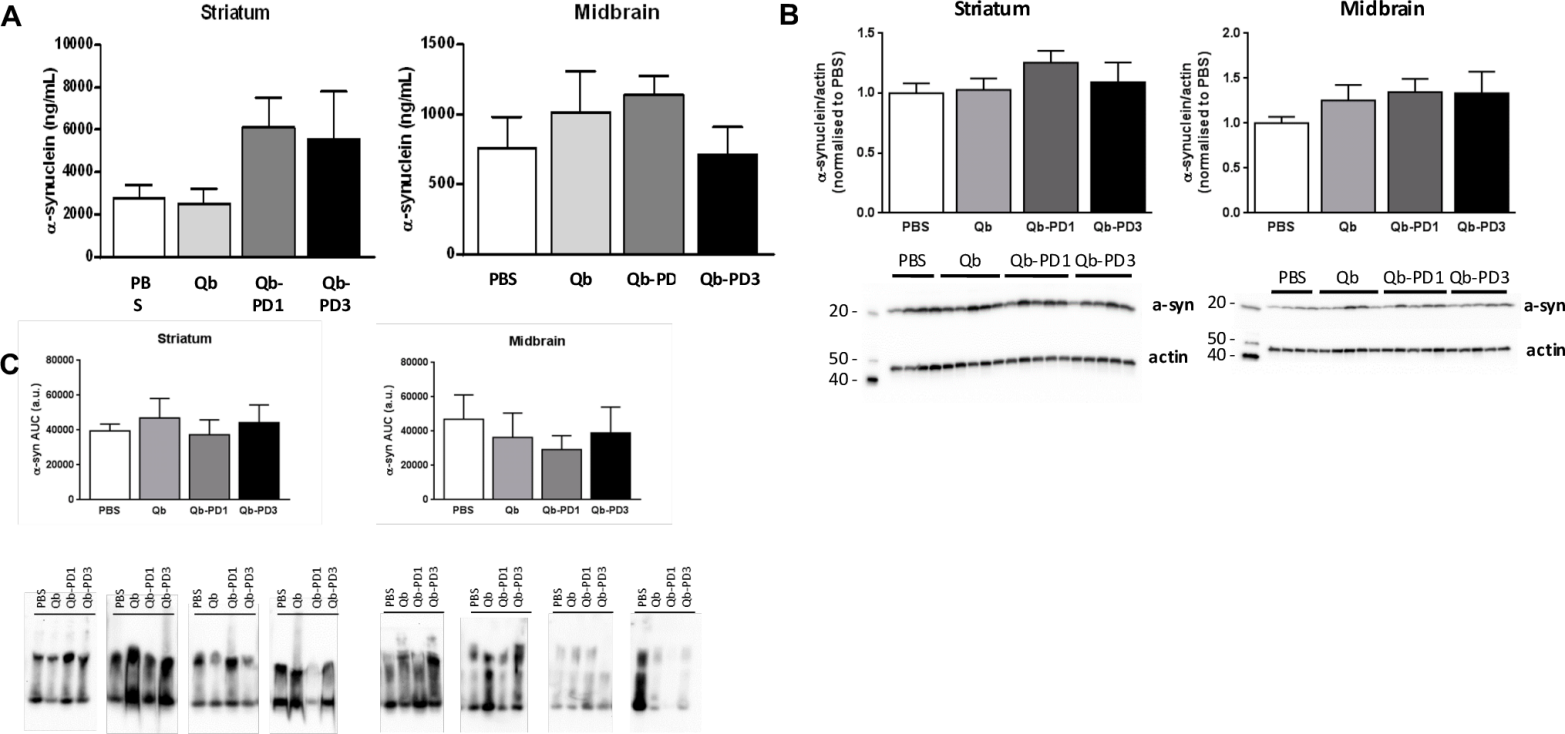
Assessment of a-syn levels following short-term immunisation. Male and female *SNCA*-OVX mice received 20 μg of Qb, Qb-PD1, or Qb-PD3, or PBS every two weeks for a month, followed by monthly injections for 1 or 2 months (total duration of immunisation: 2 or 3 months) and effects of Qb-PD vaccines on total a-syn protein levels in *SNCA*-OVX mice were examined. Striatum and midbrain lysates were prepared and 20 μg of protein was loaded per lane. Alpha-synuclein protein levels were measured by (A) ELISA, and WB following (B) SDS-PAGE and (C) native PAGE. Data are expressed as mean ± SEM (n = 3–4 mice per group) and were analysed using one-factor analysis of variance (ANOVA).

Next, we explored whether immunisation with Qb-PD1 and Qb-PD3 candidate vaccines could modify a-syn levels. One measure of Qb-PD vaccine efficacy was defined as their ability to decrease a-syn protein levels in the striatum and substantia nigra of the brain following immunisation. Since a-syn oligomers are thought to be one of the toxic forms of a-syn [42], we examined whether vaccination would decrease a-syn oligomers in the brain of Qb-PD treated mice. Measurements of total a-syn protein levels were carried out by ELISA and WB after short-term immunisation protocols (studies 1 and 2). Monomeric and oligomeric species of a-syn were examined using WB of SDS-PAGE and native PAGE, respectively. Following 2 months of circulating antibodies, we found no significant differences in a-syn protein levels in the substantia nigra or striatum of Qb-treated SNCA-OVX mice, as assessed by ELISA or WB **(Fig 6A and 6B)**. Results from native PAGE after 3 months of circulating antibodies also failed to indicate any evidence towards a decrease of a-syn oligomers **(Fig 6C)**. Finally, antibody responses induced by Qb-PD1 and Qb-PD3 vaccines were assessed for their ability to reduce levels of oligomeric a-syn by an a-syn proximity ligation assay (AS-PLA) in comparison to control animals. Results taken from the sampled brain regions demonstrated no statistically significant difference between treatment groups for the regions investigated **(S3 Fig)**. Like WB and ELISA analyses, AS-PLA examines total oligomer load and is not able to unambiguously discriminate between intracellular and extracellular forms, making it challenging to discern potential benefits. Overall, these results demonstrate that short-term immunisation was not effective in reducing oligomeric a-syn protein levels.

Previous studies using active immunisation demonstrated an effect on a-syn protein levels only after 6 to 8 months of biweekly to monthly subcutaneous administration [26, 27, 43], similar to passive immunisation protocols that provided evidence for a decrease in a-syn protein levels after 6 months of weekly administration of 10 mg/kg of monoclonal antibodies [44, 45]. We therefore investigated the effects of Qb-PD vaccines in mice immunised for 13 months.

### Behavioural effects of long-term immunisation with Qb-PD vaccines in *SNCA*-OVX mice

**Fig 7:**
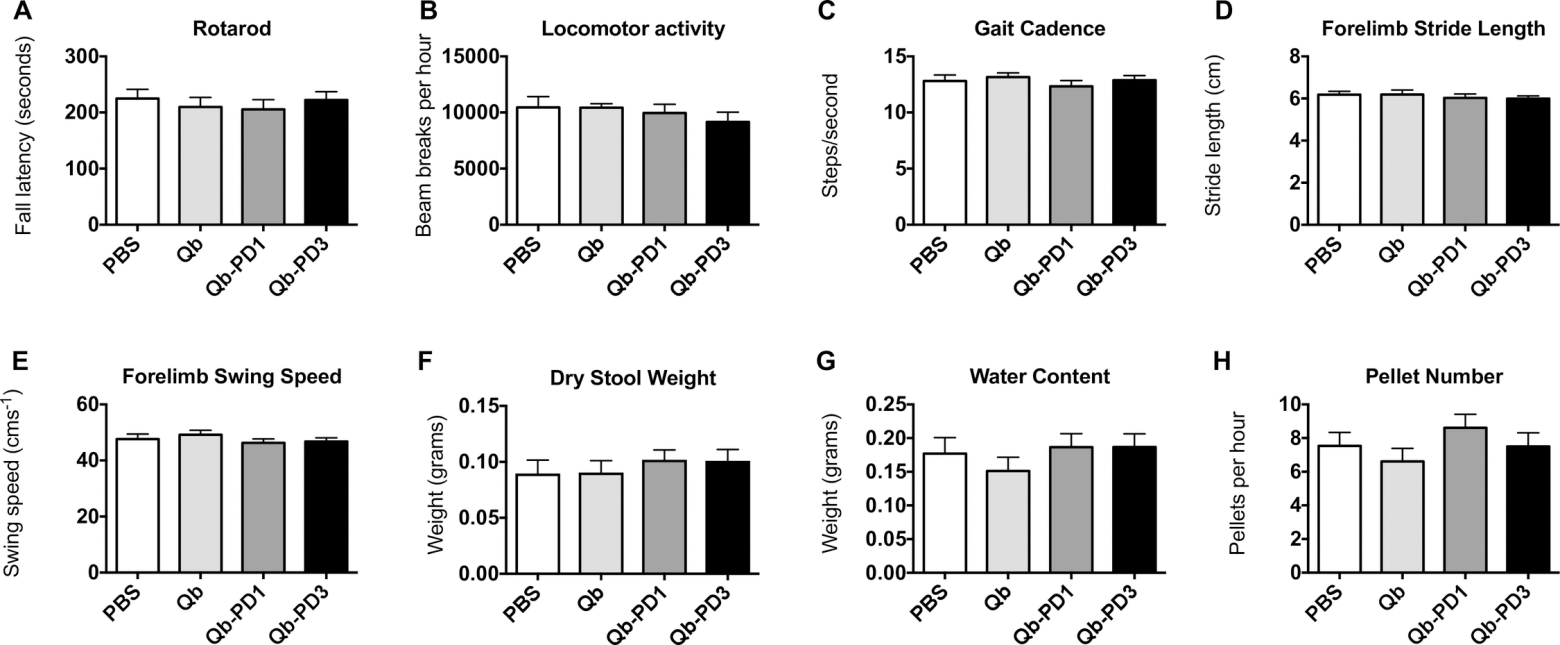
Assessment of behavioural effects of Qb-PD vaccines following long-term immunisation. Male and female *SNCA*-OVX mice received 20 μg of Qb, Qb-PD1, or Qb-PD3, or PBS every two weeks for a month, followed by monthly injections for 12 months (total duration of immunisation: 13 months). Parameters examined were (A) Rotarod fall latency, (B) spontaneous locomotor activity, (C) gait cadence, (D) forelimb stride length, (E) forelimb swing speed, (F) dry stool weight, (G) stool water content and (H) stool pellet number. Data are expressed as mean ± SEM (n = 13–16 mice per group).

Behavioural tests in 18-month old *SNCA*-OVX mice previously demonstrated motor and non-motor deficits, which included reduced fall latency on an accelerating rotating rod (Rotarod), reduced stride length, and altered gastrointestinal function [34]. Assuming that extracellular a-syn is implicated in disease symptoms in the present model, then long-term immunisation would be expected to lead to differences in motor and/or non-motor phenotypes between treatment groups. We therefore sought to examine behavioural sequelae of a-syn immunisation. Motor coordination as measured by Rotarod fall latency **(Fig 7A)**, locomotor activity **(Fig 7B)**, and gait parameters including cadence **(Fig 7C)**, stride length **(Fig 7D)**, limb swing speed **(Fig 7E)** were not different between treatment groups. Forelimb parameters are presented here, and are indicative of hindlimb data (not shown). There was no difference in muscle strength between treatment groups, as assessed by the Kondziela’s inverted screen test. Gastrointestinal dysfunction is a common non-motor symptom in PD, and can manifest before motor symptoms became apparent [46]. For this reason, we examined the effect of immunisation against a-syn on gastrointestinal function. We found no difference between treatment groups in any of the parameters tested, including dry stool weight **(Fig 7F)**, water content **(Fig 7G)** or pellet number **(Fig 7H)**.

### Molecular effects of long-term immunisation with Qb-PD vaccines in *SNCA*-OVX mice

**Fig 8:**
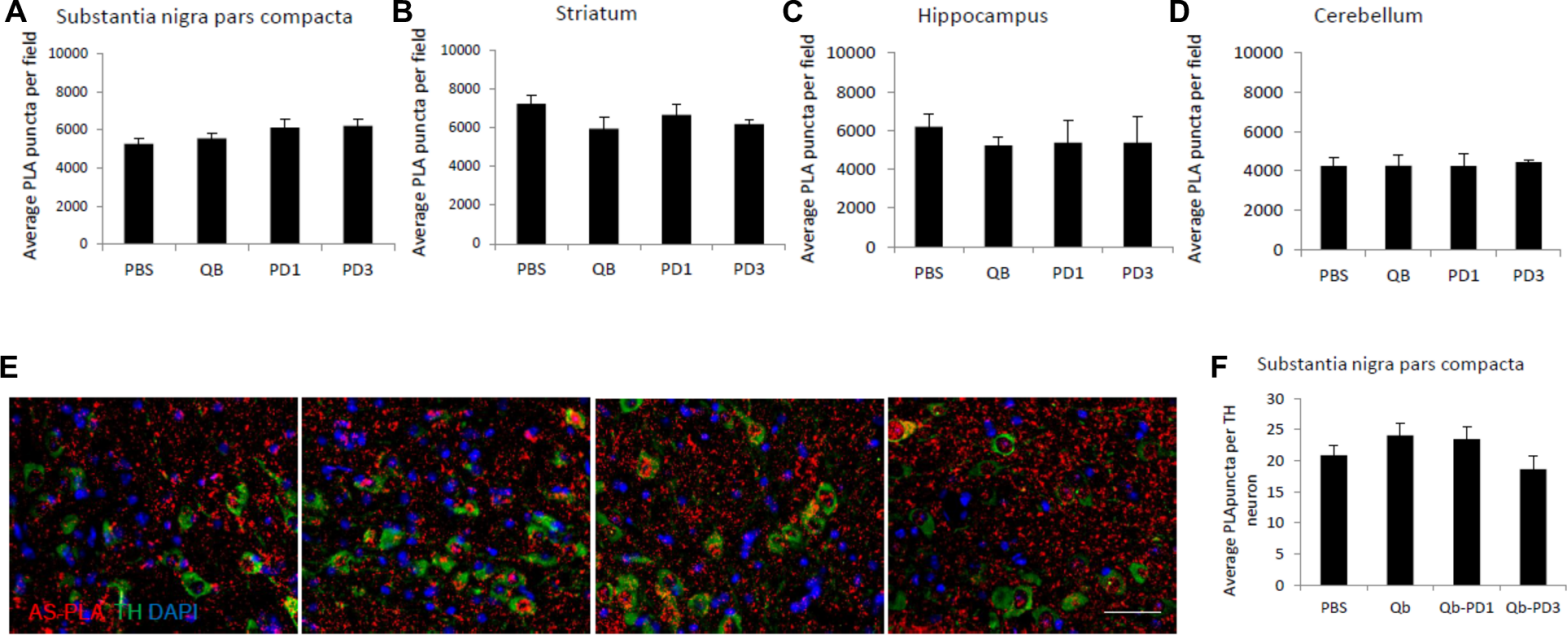
Effects of long-term immunisation with Qb-PD vaccines on a-syn aggregates. Male and female *SNCA*-OVX mice received 20 μg of Qb, Qb-PD1, Qb-PD3 or PBS every two weeks for a month, followed by monthly injections for 12 months (total duration of immunisation: for 13 months). (A-D) Immunofluorescence analysis was performed to detect a-syn aggregates with PLA in the substantia nigra, striatum, hippocampus and cerebellum. PLA puncta were quantified using ImageJ. (E) PLA puncta in 26–36 TH positive neurons were quantified and expressed as average mean of puncta per positive cell ± SEM (n = 3–4 mice per group), ANOVA followed by Dunn’s post hoc test. Scale bar 50 μm.

Since molecular changes can precede behavioural manifestation, we investigated whether a-syn aggregation was reduced by vaccination using AS-PLA analysis in several regions of the brain. AS-PLA analysis revealed no changes for any of the treatment groups in the substantia nigra, striatum, hippocampus and cerebellum **(Fig 8A–8D)**. We hypothesised that subtle changes might be observed in different cellular populations and tested whether vaccination would induce changes in a-syn oligomeric content of TH-positive cells **(Fig 8E)**. No significant changes between any of the studied groups were revealed. The absence of any molecular and behavioural phenotypes urged us to examine whether the antibodies were indeed interacting with a-syn in the brain. To this end, using immunofluorescence, we verified that putative alpha-synuclein aggregates, immune complexes and microglia co-localised. We surmised that if aggregates containing antibodies and a-syn that were engulfed by microglia were detected, we may be able to detect differences in the immune activity between vaccinated groups. Even though all groups presented putative a-syn immune complexes within microglial cells, no significant differences were detected in short- and long-term immunised animals **(S4 and S5 Figs)**. We also evaluated expression of MHCII and Fc-gamma receptor as an indication of microglial activation or recruitment; however, western blotting showed no differences in long-term immunised animals (**S6C Fig**). Taken altogether these results suggest that immunisation with Qb-PD1 and Qb-PD3 vaccines was not able to elicit an effect on a-syn levels in *SNCA*-OVX mice.

## Discussion

The results of the present investigation demonstrate that vaccines based on Qb VLPs and targeting a-syn (Qb-PD1 and Qb-PD3) induce antibodies that selectively recognise oligomeric and aggregated a-syn, while showing poor affinity for soluble monomeric a-syn. This selectivity is a result of increased avidity for aggregate species which could be favourable in an immunotherapeutic context as the antibodies would likely avoid neutralising physiological monomeric a-syn. These vaccine candidates are safe and well-tolerated in the immunisation protocols tested. In the *SNCA*-OVX transgenic mouse model of Parkinson’s disease, immunisation with Qb-PD vaccines is, however, not able to improve motor or non-motor symptoms of the disease.

Successful generation of novel vaccines targeting a-syn, in particular towards the C-terminus region, demonstrated that induced antibodies show preference for oligomeric a-syn species. Although the ELISA results demonstrated that the antibodies recognised monomeric protein, at least if coated on plastic, competition studies in solution revealed that both Qb-PD1 and Qb-PD3 vaccines induced antibodies with a strong (at least 100-fold) preference for oligomeric species of a-syn. It is therefore unlikely that the affinities of the generated antibodies for the Qb-PD vaccines would effectively intercept monomeric species in solution. Parameters indicating preclinical safety were satisfactory and long-term immunisation of mice was well tolerated, with no vaccine-mediated adverse effects. This agrees with previous studies in our laboratory, where VLP vaccines consistently induced B-cells to generate humoral responses, in particular to self-antigens [47]. VLPs are also particularly useful for antigens that typically exhibit low immunogenicity, such as peptides [32]. It is significant that high antibody titres were achieved without the need for additional adjuvants (e.g. alum), which can often be a requirement for alternative platforms [48] and represents a distinct advantage over other systems that typically require additional strong adjuvants. Our results indicate that the level of antibodies generated with candidate Qb-PD vaccines matched those obtained by a bioequivalent to an AD vaccine (CAD106), that similarly used a short peptide. Those levels of antibodies were able to reduce amyloid beta plaques in preclinical murine model of AD [32]. Based on our findings, Qb-PD3 had some advantages over Qb-PD1, although both vaccines were able to generate antibodies that successfully detect their respective peptides as well as oligomeric full-length and recombinant proteins. Levels of antibodies induced by Qb-PD3, particularly in their recognition of oligomeric and aggregated a-syn, was markedly better than Qb-PD1.

This emphasises an important outcome from the study which was the generation of a vaccine which induced antibodies with a clear preference for oligomeric and aggregated species of a-syn. Designs for the vaccines included peptides that targeted the extremities at both ends of the molecule, as it was hypothesised that these regions would be the most accessible in aggregates. It was interesting to note that antibodies directed towards the N-terminus did not detect a-syn in mouse brain sections nor human post-mortem brain, suggesting that the amphipathic region of a-syn is occluded or possibly that the affinity of antibodies was too low. The strongest responses were found to be directed towards the C-terminus of a-syn. The acidic charge present there may confer greater hydrophilicity, allowing this region to be more solvent exposed. This in turn could explain the ability of small and intermediate aggregates to maintain solubility and would impart increased mobility of these molecules into extracellular spaces, contributing to their capacity to spread. In agreement with these observations, antibodies targeting the carboxy-terminus of a-syn were shown to reduce cell-to-cell propagation of a-syn in vitro [44], as well as reducing neuronal and glial accumulation of a-syn, attenuating synaptic and axonal pathology and improving behavioural deficits in several investigations using mouse models over-expressing a-syn [26, 27, 43–45, 49]. However, two separate studies have demonstrated efficacy of immunotherapies based on antibodies recognising epitopes within the N-terminus of a-syn. In the first one, WT mice were inoculated with a-syn preformed fibrils in the striatum, followed by intraperitoneal administration of Syn 303 monoclonal antibodies (aa 1–5 peptide specific). In these mice, passive immunisation was able to reduce the spread of pathological a-syn, rescue dopaminergic neuronal loss in the substantia nigra and associated motor deficits induced by transmission of pathological a-syn (grip strength test) [23]. In the second study, peptide specific antibodies against the N-terminus of a-syn (aa 16–35) protected against dopaminergic cell death and ameliorated behavioural deficits in an adeno-associated virus (AAV) a-syn rat model of PD [50]. This would suggest targeting the N-terminus or C-terminus region of a-syn might represent a useful immunotherapy approach to treat PD as well as other synucleinopathies. Although in our studies antibodies to the N-terminus of a-syn failed to recognise Lewy bodies or neurites from human PD brain samples.

Due to the high degree of similarity between a-syn and b-syn at the carboxyl-end, antibodies from vaccine Qb-PD3 also cross-reacted with b-syn in vitro **(S7 Fig)**. Cross-reactivity with b-syn represents a potentially undesirable outcome. However, unlike a-syn, b-syn does not appear in Lewy bodies and is only found as monomers. Previous work has suggested a role for b-syn to interact with a-syn in circumstances where a-syn levels are high, thereby acting as chaperone to regulate a-syn self-association [51, 52]. As mentioned above, the generated antibodies are unlikely to bind to monomeric a-syn in solution due to their low affinity, and therefore are equally unlikely to interact with monomeric b-syn. Interestingly, antibodies from Qb-PD1 treated mice did not recognise b-syn. Should there still be cause for concern, modifying vaccine design by panning through short sequences between the two regions represented by PD1 and PD3 should maximise antibody response but minimise cross-reactivity.

The encouraging biochemical characterisation of Qb-PD vaccine responses were not matched by efficacy analyses in vivo. Whereas the transgenic *SNCA*-OVX mouse may represent a useful model to study the biological effects of a-syn oligomers, it may not lend itself to assessment of antibody-mediated therapeutic interventions as attempted in the present project for the following reasons: (i) as our PLA data suggest, a-syn expression manifests intracellularly, leading to the widespread occurrence of oligomers *inside* all affected cells which are therefore unable to be directly engaged by *extra*cellular antibodies. (ii) As all affected neurons express the *SNCA* transgene, pathology will arise within all cells without the requirement for the spread of pathological protein. In this case there may be little to be gained by intercepting the spread of putative aggregate seeding oligomers. These points would go some way towards explaining the inability to effect changes to the gross bioburden of a-syn and a-syn oligomers already present within cells. Hence, the model used here may not show the slow spread of oligomeric a-syn along axonal pathways as seen in PD patients. (iii) Finally, levels of antibodies inside the CNS may not be not be sufficient to effectively remove oligomers.

The inability to reduce a-syn burden may be explained by the inability of the methods used to discriminate between levels of intra- and extra-cellular oligomers. The whole-cell assays to determine bioburden were unable to detect differences between the treatment groups. Interestingly, the CSF from patients with PD or dementia with PD was shown to contain higher levels of oligomeric a-syn compared to healthy controls [22, 53]. Therefore, analysis of CSF of mice following vaccination may represent a more appropriate sample to observe differences in extracellular a-syn bioburden and potential effects of Qb-PD vaccination.

Consequently, repeating the study in a preclinical mouse model that more accurately reconstitutes a-syn-mediated spreading of aggregated forms (in particular oligomeric a-syn but potentially others as well) could prove useful. Possible approaches include intra-striatal injection of recombinant a-syn oligomers or preformed fibrils, as well as injections of purified Lewy body extracts from human post mortem brains to seed aggregates in one area and observe whether vaccination can restrict spreading and associated neurotoxicity. Mouse models using intrastriatal or intramuscular injections of a-syn preformed fibrils in transgenic and WT mice have shown a neurodegenerative cascade characterised by widespread a-syn inclusion pathology in the CNS, selective loss of dopaminergic neurons, neuro-inflammation (astrogliosis and microgliosis) and motor symptoms [17, 18, 54, 55]. In these models, a single administration of a-syn prefibrils was able to initiate PD-like inclusions and transmit disease in vivo. Other mouse models of interest based on cell-to-cell transmission of a-syn include those using post mortem brain homogenates from patients with synucleinopathies (DLB, MSA and PD) [54, 56, 57].

Investigation of potential changes in neuro-inflammatory status by monitoring microglia activation would be of interest, as a-syn oligomers have been shown to stimulate activation via Toll-like receptor 2 (TLR2) [58]. Therefore, examination of the effects of candidate vaccines on the proportion of microglia with upregulated MHCII on the cell surface (an indication of microglial activation) might reveal differences in the level of intracerebral oligomers. Recent reports also indicate that regulatory T cell infiltrates play an active role in modulating neuroinflammatory status [59]. Caution is however warranted with such approaches. Adverse effects such as meningo-encephalitis observed with the first-generation of vaccines developed for the treatment of Alzheimer’s disease (AD) clearly provided evidence of the dangers of inducing cytotoxic lymphocytes to self-antigens [60–63]. The lessons uncovered in the pursuit of AD immunotherapies support the rationale of using short peptides to avoid antigen-specific cytotoxic responses when targeting self-antigens, particularly in the brain [64], as implemented in the present investigation. An alternative is represented by the use of mimetic epitopes (“mimotopes”) that are not recognised as self-antigens [65].

## Conclusions

We have shown that vaccines based on Qb VLPs and targeting a-syn (Qb-PD1 and Qb-PD3) induce antibodies that selectively recognise oligomeric and aggregated a-syn, while showing poor affinity for soluble monomeric a-syn these vaccines show promise. However, further preclinical work using a relevant PFF spread mouse model and detailed analyses of cellular immune responses is required to further explore the potential of Qb-PD vaccines as novel therapies for synucleinopathies.

## Supporting information

S1 Fig Peptide-specific antibody responses induced by Qb-PD vaccines. Male and female *SNCA*-OVX mice received 20 μg of Qb, Qb-PD1, Qb-PD3 or PBS subcutaneously at d0, d14, d28 and monthly thereafter for studies 1 to 4. Antibody titres were determined using ELISA and are expressed as mean ± SEM (n = 4–6 mice per group for studies 1–3 and n = 13–16 mice per group for study 4).
(TIF)

S2 Fig Recognition of a-syn in mouse brain tissue. Immunofluorescence on free-floating sections from the substantia nigra of 3-month-old *SNCA*-OVX mice. Primary antibodies were purified IgGs (1 mg/mL) of vaccinated mice used at 1:250 and rabbit anti-TH at 1:500 (Millipore). Secondary antibodies were Alexa Fluor anti-mouse 488 nm and goat anti-rabbit 594 nm. Scale bar, 50 μm.
(TIF)

S3 Fig Safety and tolerability of Qb-PD vaccines following 2-month subcutaneous immunisation. Male and female *SNCA*-OVX mice received 20 μg of Qb, Qb-PD1, Qb-PD3 or PBS every two weeks for a month, followed by monthly injections for a month (total duration of immunisation: 2 months). Parameters examined were (A) body weight, (B) spleen weight and (C) histology of spleen. Data are expressed as mean ± SEM (n = 5–6 mice per group) and were analysed using two-factor (A) or one-factor (B) analyses of variance (ANOVA). RP, red pulp, WP, white pulp. Scale bar, 100 μm.
(TIF)

S4 Fig Assessment of a-syn levels following short term immunisation. Male and female *SNCA*-OVX mice received 20 μg of Qb, Qb-PD1, Qb-PD3 or PBS every two weeks for a month, followed by monthly injections for 3 months (total duration of immunisation: 4 months). (A) Effects of Qb-PD vaccines on a-syn oligomers levels were examined using brightfield AS-PLA. (B) Representative image of a-syn oligomeric puncta in the striatum of *SNCA*-OVX mice. Data are expressed as mean ± SEM (n = 4 mice per group) and were analysed using a one-factor ANOVA followed by post hoc Dunn’s test. SNc, substantia nigra. Scale bar, 100 μm.
(TIF)

S5 Fig Assessment of putative a-syn/antibody complexes internalised by microglia after short-term immunisation. Male and female *SNCA*-OVX mice received 20 μg of Qb, Qb-PD1, Qb-PD3 or PBS every two weeks for a month, followed by monthly injections for 1–2 months (total duration of immunisation: 2–3 months). (A) Immunofluorescence analysis was performed to detect a-syn complexes (aggregated punctate green stain), immune complexes detecting IgG (grey) and microglia (Iba1 red stain) in the substantia nigra to determine whether these were affected by vaccination. (B) Data are expressed as mean of four quantified fields ± SEM (n = 3–4 mice per group) and were analysed using a one-factor ANOVA followed by post hoc Dunn’s test. Scale bar, 50 μm.
(TIF)

S6 Fig Assessment of putative a-syn/antibody complexes internalised by microglia after long-term immunisation. Male and female *SNCA*-OVX mice received 20 μg of Qb, Qb-PD1, Qb-PD3 or PBS every two weeks for a month, followed by monthly injections for 12 months (total duration of immunisation: 13 months). (A) Immunofluorescence analysis was performed to detect a-syn complexes (aggregated punctate green stain), immune complexes detecting IgG (grey) and microglia (Iba1 red stain) in the substantia nigra to determine whether these were affected by vaccination. (B) Data are expressed as mean of four quantified fields ± SEM (n = 3–5 mice per group) and were analysed using a one-factor ANOVA followed by post hoc Dunn’s test. Scale bar, 50 μm. (C) WB of MHCII and CD16 (Fc-gamma receptor) of male and female *SNCA*-OVX mice that received 20 μg of Qb, Qb-PD1, Qb-PD3 or PBS every two weeks for a month, followed by monthly injections for 12 months (total duration of immunisation: 13 months). Data are expressed as mean of the antibody/actin ratio ± SEM (n = 4 mice per group) and were analysed using a one-factor ANOVA followed by post hoc Dunn’s test.
(TIF)

S7 Fig Beta-synuclein cross-reactivity of antibodies induced by immunisation with Qb-PD3 vaccine. Male and female *SNCA*-OVX mice received 20 μg of Qb, Qb-PD1, Qb-PD3 or PBS subcutaneously at d0, d14, d28 and d56. ELISA plates were coated with full-length recombinant b-syn protein. Antibody titres were determined using ELISA and are expressed as mean ± SEM (n = 6 mice per group, from studies 2 and 4).
(TIF)

S1 Checklist ARRIVE guidelines checklist. (PDF)
