# Supplementary figures and images for: Preclinical development of a vaccine against oligomeric alpha-synuclein based on virus-like particles
Source: PLoS One. 2017 Aug 10;12(8):e0181844. doi: 10.1371/journal.pone.0181844 (PMC5552317; doi:10.1371/journal.pone.0181844)

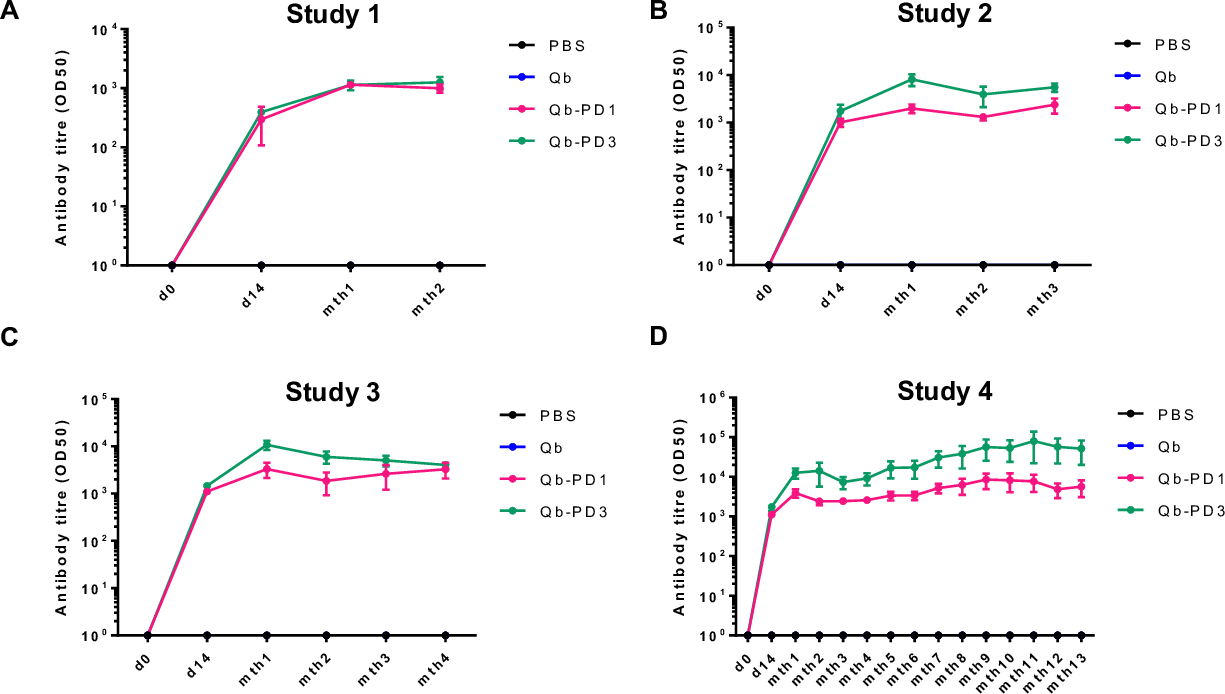

Supplement: S1 Fig — Male and female SNCA-OVX mice received 20 μg of Qb, Qb-PD1, Qb-PD3 or PBS subcutaneously at d0, d14, d28 and monthly thereafter for studies 1 to 4. Antibody titres were determined using ELISA and are expressed as mean ± SEM (n = 4–6 mice per group for studies 1–3 and n = 13–16 mice per group for study 4). (TIF) [file pone.0181844.s001.tif]

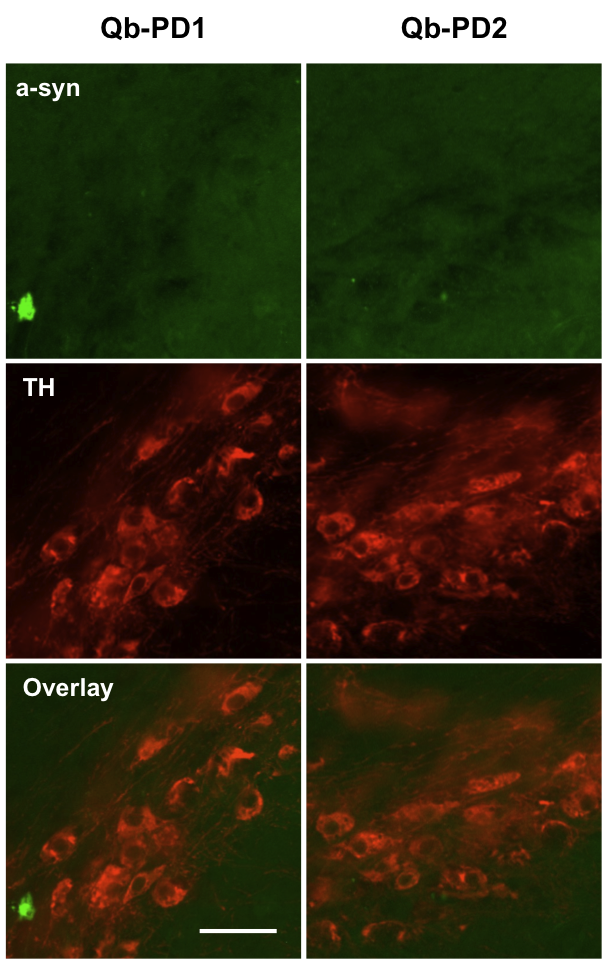

Supplement: S2 Fig — Immunofluorescence on free-floating sections from the substantia nigra of 3-month-old SNCA-OVX mice. Primary antibodies were purified IgGs (1 mg/mL) of vaccinated mice used at 1:250 and rabbit anti-TH at 1:500 (Millipore). Secondary antibodies were Alexa Fluor anti-mouse 488 nm and goat anti-rabbit 594 nm. Scale bar, 50 μm. (TIF) [file pone.0181844.s002.tif]

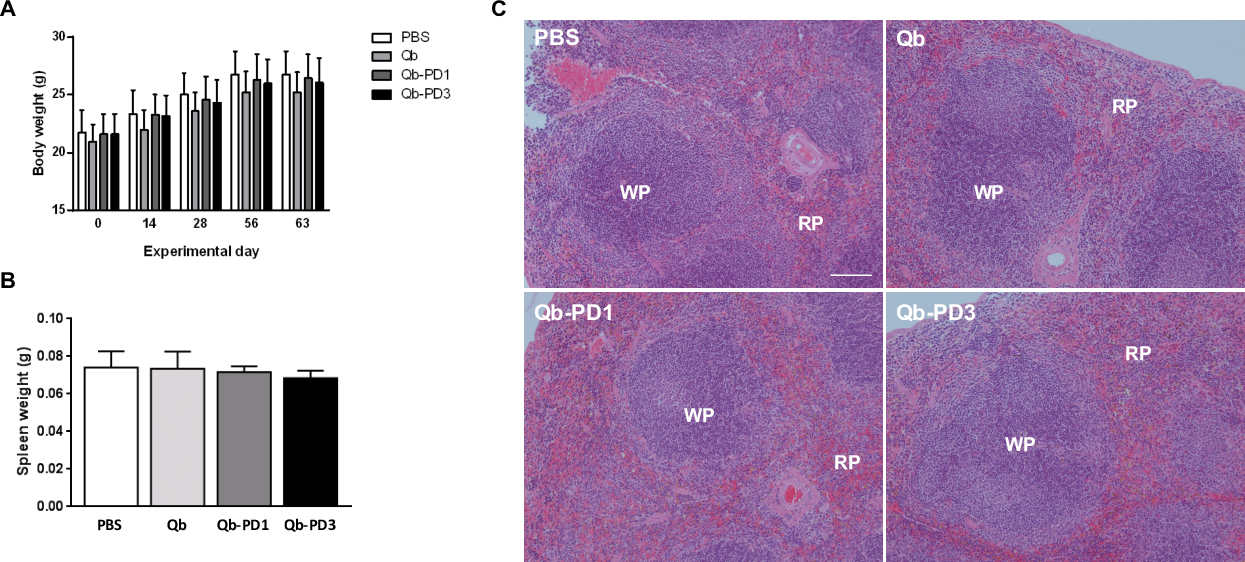

Supplement: S3 Fig — Male and female SNCA-OVX mice received 20 μg of Qb, Qb-PD1, Qb-PD3 or PBS every two weeks for a month, followed by monthly injections for a month (total duration of immunisation: 2 months). Parameters examined were (A) body weight, (B) spleen weight and (C) histology of spleen. Data are expressed as mean ± SEM (n = 5–6 mice per group) and were analysed using two-factor (A) or one-factor (B) analyses of variance (ANOVA). RP, red pulp, WP, white pulp. Scale bar, 100 μm. (TIF) [file pone.0181844.s003.tif]

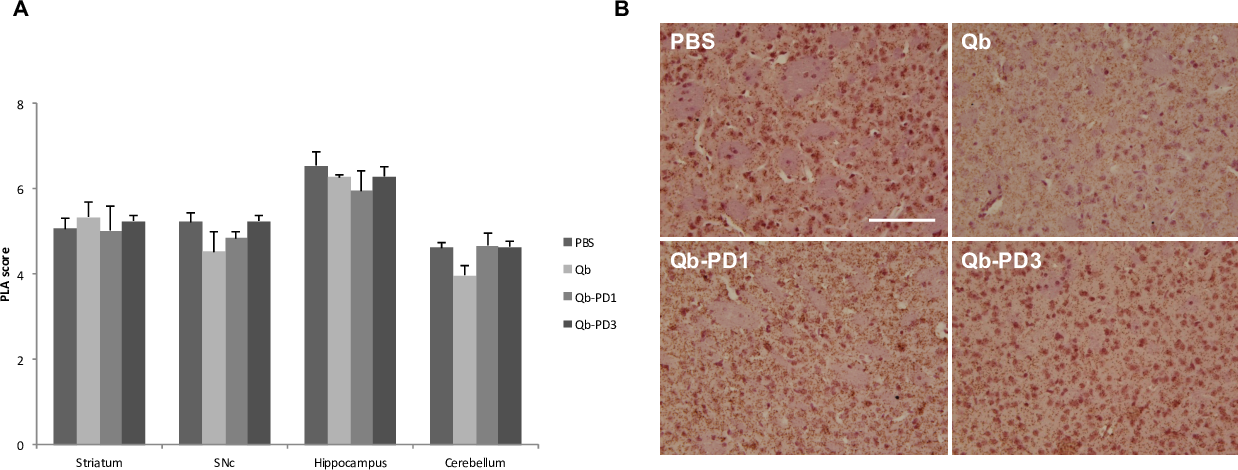

Supplement: S4 Fig — Male and female SNCA-OVX mice received 20 μg of Qb, Qb-PD1, Qb-PD3 or PBS every two weeks for a month, followed by monthly injections for 3 months (total duration of immunisation: 4 months). (A) Effects of Qb-PD vaccines on a-syn oligomers levels were examined using brightfield AS-PLA. (B) Representative image of a-syn oligomeric puncta in the striatum of SNCA-OVX mice. Data are expressed as mean ± SEM (n = 4 mice per group) and were analysed using a one-factor ANOVA followed by post hoc Dunn’s test. SNc, substantia nigra. Scale bar, 100 μm. (TIF) [file pone.0181844.s004.tif]

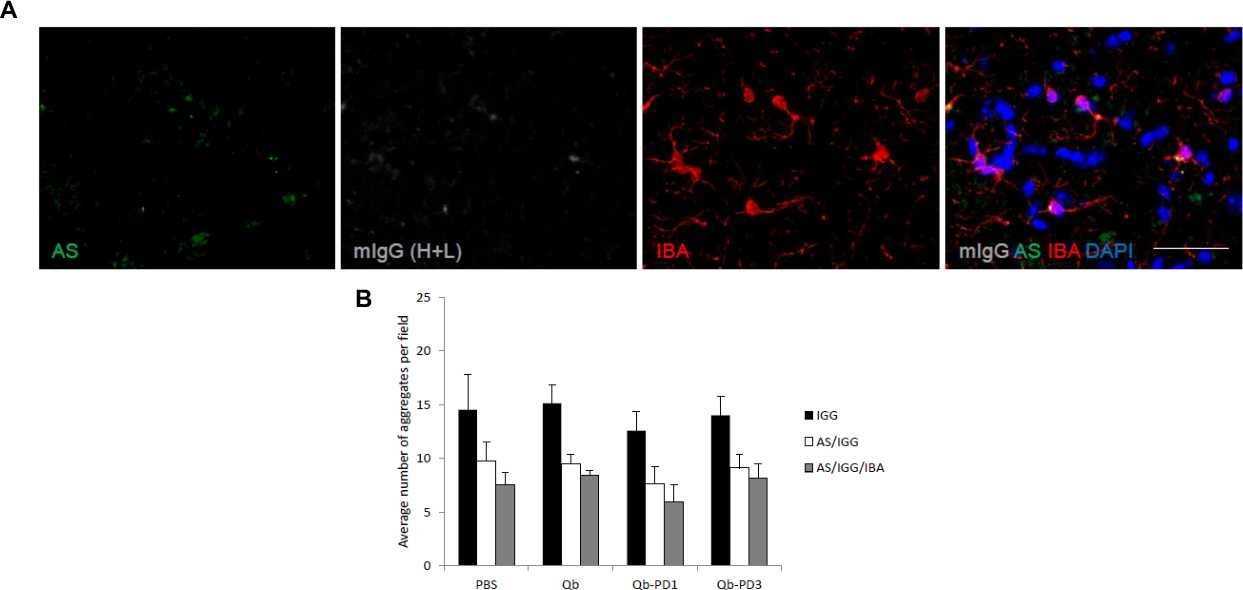

Supplement: S5 Fig — Male and female SNCA-OVX mice received 20 μg of Qb, Qb-PD1, Qb-PD3 or PBS every two weeks for a month, followed by monthly injections for 1–2 months (total duration of immunisation: 2–3 months). (A) Immunofluorescence analysis was performed to detect a-syn complexes (aggregated punctate green stain), immune complexes detecting IgG (grey) and microglia (Iba1 red stain) in the substantia nigra to determine whether these were affected by vaccination. (B) Data are expressed as mean of four quantified fields ± SEM (n = 3–4 mice per group) and were analysed using a one-factor ANOVA followed by post hoc Dunn’s test. Scale bar, 50 μm. (TIF) [file pone.0181844.s005.tif]

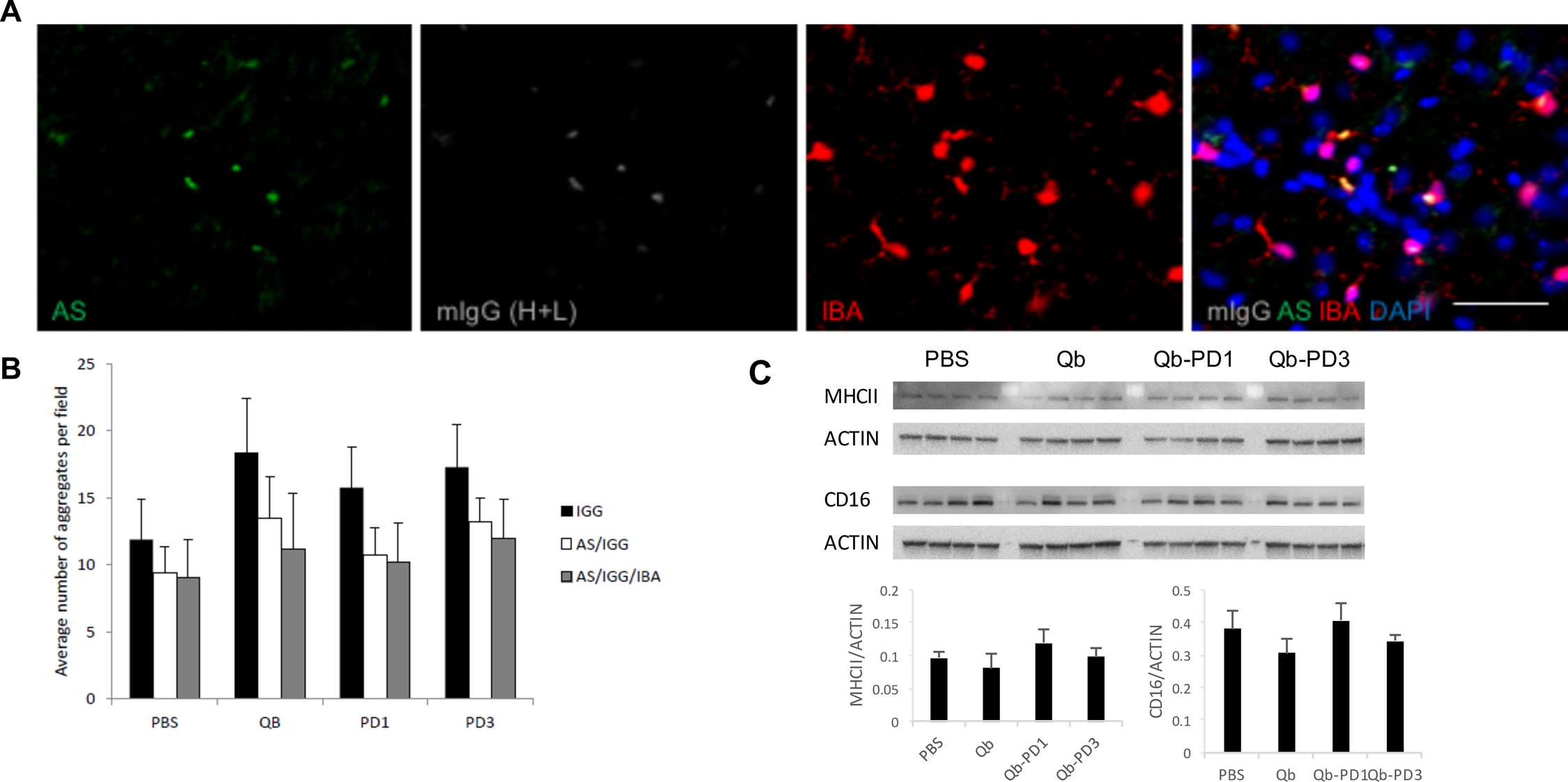

Supplement: S6 Fig — Male and female SNCA-OVX mice received 20 μg of Qb, Qb-PD1, Qb-PD3 or PBS every two weeks for a month, followed by monthly injections for 12 months (total duration of immunisation: 13 months). (A) Immunofluorescence analysis was performed to detect a-syn complexes (aggregated punctate green stain), immune complexes detecting IgG (grey) and microglia (Iba1 red stain) in the substantia nigra to determine whether these were affected by vaccination. (B) Data are expressed as mean of four quantified fields ± SEM (n = 3–5 mice per group) and were analysed using a one-factor ANOVA followed by post hoc Dunn’s test. Scale bar, 50 μm. (C) WB of MHCII and CD16 (Fc-gamma receptor) of male and female SNCA-OVX mice that received 20 μg of Qb, Qb-PD1, Qb-PD3 or PBS every two weeks for a month, followed by monthly injections for 12 months (total duration of immunisation: 13 months). Data are expressed as mean of the antibody/actin ratio ± SEM (n = 4 mice per group) and were analysed using a one-factor ANOVA followed by post hoc Dunn’s test. (TIF) [file pone.0181844.s006.tif]

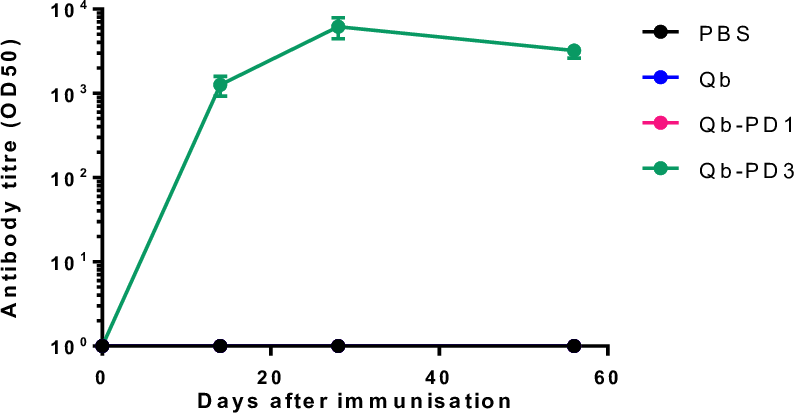

Supplement: S7 Fig — Male and female SNCA-OVX mice received 20 μg of Qb, Qb-PD1, Qb-PD3 or PBS subcutaneously at d0, d14, d28 and d56. ELISA plates were coated with full-length recombinant b-syn protein. Antibody titres were determined using ELISA and are expressed as mean ± SEM (n = 6 mice per group, from studies 2 and 4). (TIF) [file pone.0181844.s007.tif]
